# Stochastic nature and physiological implications of 5′-NAD RNA cap in bacteria

**DOI:** 10.1093/nar/gkae813

**Published:** 2024-09-26

**Authors:** Jana Wiedermannová, Ravishankar Babu, Yulia Yuzenkova

**Affiliations:** Centre for Bacterial Cell Biology, Biosciences Institute, Faculty of Medical Sciences, Newcastle University, Newcastle upon Tyne NE2 4AX, UK; Institute of Microbiology of the Czech Academy of Sciences, Vídeňská 1083, 142 20 Prague, Czech Republic; Centre for Bacterial Cell Biology, Biosciences Institute, Faculty of Medical Sciences, Newcastle University, Newcastle upon Tyne NE2 4AX, UK; Centre for Bacterial Cell Biology, Biosciences Institute, Faculty of Medical Sciences, Newcastle University, Newcastle upon Tyne NE2 4AX, UK

## Abstract

RNA 5′-modification with NAD^+^/NADH (oxidized/reduced nicotinamide adenine dinucleotide) has been found in bacteria, eukaryotes and viruses. 5′-NAD is incorporated into RNA by RNA polymerases (RNAPs) during the initiation of synthesis. It is unknown (i) which factors and physiological conditions permit substantial NAD incorporation into RNA *in vivo* and (ii) how 5′-NAD impacts gene expression and the fate of RNA in bacteria. Here we show in *Escherichia coli* that RNA NADylation is stimulated by low cellular concentration of the competing substrate ATP, and by weakening ATP contacts with RNAP active site. Additionally, RNA NADylation may be influenced by DNA supercoiling. RNA NADylation does not interfere with posttranscriptional RNA processing by major ribonuclease RNase E. It does not impact the base-pairing between RNAI, the repressor of plasmid replication, and its antisense target, RNAII. Leaderless NADylated model mRNA *cI-lacZ* is recognized by the 70S ribosome and is translated with the same efficiency as triphosphorylated *cI-lacZ* mRNA. Translation exposes the 5′-NAD of this mRNA to de-capping by NudC enzyme. We suggest that NADylated mRNAs are rapidly degraded, consistent with their low abundance in published datasets. Furthermore, we observed that ppGpp inhibits NudC de-capping activity, contributing to the growth phase-dependency of NADylated RNA levels.

## Graphical Abstract

**Graphical Abstract:**
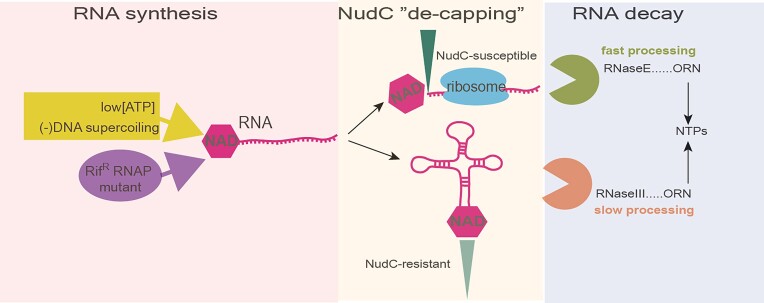

## Introduction

Metabolic cofactors, including NAD, FAD and other analogues of ADP, GDP and UDP, were found as 5′-terminal modifications of RNA in bacteria, archaea, eukaryotes and viruses (1–4). 5′-RNA NADylation is the most abundant and best-studied modification of this type. Various unrelated RNA polymerases, including single- and multi-subunit RNA polymerases and primases, install NAD on RNA using it as an initiation substrate for RNA synthesis (5,6). The 5′-NAD bears a superficial resemblance to the classic m^7^G cap found in eukaryotes and is sometimes regarded as a non-canonical RNA cap.

Notably, the identity and NADylation status of various RNA species vary considerably depending on the 5′-NAD RNA quantification method—NAD captureSeq (1), NAD tagSeq (7) or mass spectrometry analysis (2,8). The majority of highly NADylated RNAs in *Escherichia coli* are small regulatory RNAs (1,6) that function via base-pairing with a target RNA, mRNAs or other sRNAs. For instance, RNAI base-pairs with RNAII, to regulate replication of the plasmids with ColE1 origin, widespread among γ-proteobacteria (9), Sib RNAs base-pair with their corresponding antisense Ibs RNAs (10) and GcvB regulates expression of a high number of target genes by base-pairing with their mRNAs (11). Nonetheless, apart from sRNAs, a number of 5′-NADylated mRNA species were detected in *E. coli* by Zhang *et al.* (7).

Under standard growth conditions, ATP is present in more than three times higher concentrations than NAD (12). ATP was shown to compete directly with NAD as the initiating substrate *in vitro*. In these *in vitro* experiments, it was found that apparent *K_M_* is lower for ATP vs NAD: ∼100 versus ∼400 μM (5) and the specificity constant (*V*_max_/*K_M_*) illustrates RNAP preference for ATP incorporation over NAD (6). It is not surprising therefore, that only a certain portion of a given RNA is found to be NADylated. The highest NADylated species in *E. coli* are genomic SibD, SibE and SibC small RNAs (71.3, 55.2 and 51.5% respectively (7)) and RNAI, 26% (1). Predictably, NADylation increases with the increase of cellular concentration of NAD precursor, nicotinamide mononucleotide (NMN) (13). What the minimal concentration of NAD is to allow capping and how NAD ‘wins over’ the abundant ATP is not known. It is also unknown if the extent of NADylation reflects some specific conditions or RNAP properties and whether RNA could be 100% NADylated. Physiological conditions which would favour NAD incorporation are not yet identified, apart from reports of higher RNA NADylation in stationary growth phase in *E. coli* (7).

By analogy with canonical m^7^G capping, de-capping (deNADing) enzymes were proposed to exist in all kingdoms (14,15). In *E. coli* the de-capping function is played by NudC, a NUDIX hydrolase (enzymes that catalyse the hydrolysis of nucleoside diphosphates linked to other moieties X) (16). In archaea, NAD-RNA might be first converted to ADPR-RNA before de-capping (4).

Considering its cap-like structure, there are expectations of 5′-NAD serving some function in RNA fate and translation. Yet, it is still majorly unknown how the 5′-NAD affects the processing of RNA in *E. coli*. So far, we rely on one experiment showing RNAI stabilized by 5′-NAD in *E. coli* in the absence of NudC (6). The mechanism of this stabilisation is unclear. RNase E, the main RNA processing enzyme of *E. coli*, cleaves RNA internally. The processing of only a minor part of RNA species by RNase E depends on the nature of their 5′ end—monophosphorylated form is cleaved much faster than triphosphorylated one (17). RNase E cleavage products are further degraded by 3′-5′ exonucleases, followed by essential oligoribonuclease Orn, the only enzyme able to degrade oligonucleotides shorter than 5 nt-long (18). If Orn tolerates ‘capped’ dinucleotides (here and further, the length of RNA is in conventional nucleotides) is not known.

Whether 5′-NAD can affect the translation of prokaryotic mRNA is unknown. Bacterial translation machinery doesn’t depend on modification at the mRNA 5′-end, unlike eukaryotic one, where the cap is essential for initiation (19). Yet in the case of a leaderless mRNA, 5′-NAD of mRNA may directly affect the assembly of the translation initiation complex, since ribosomes must bind right at the AUG start codon, which is changed to NAD-UG if RNA is NADylated. Leaderless mRNAs are found in all domains of life, widespread in bacteria, and frequent especially in *Mycobacteria, Streptococcus* and *Deinococcus* phyla (20).

Aided with our new NAD-RNA quantification method, we show which physiological and cellular factors affect NAD incorporation and to what limited extent 5′-NAD may play a function of an RNA cap, i.e. affects gene expression and the fate of mRNA in cell.

## Materials and methods

### Materials

ATP and CTP were from GE Healthcare; NAD+, NADH, NADP+, FAD, dpCoA, UDG-Glc and UDP-GlcNAc, 2-acetylbenzofuran, kasugamycin were from Sigma Aldrich; ppGpp was from Jena Bioscience.

### Strains and plasmids

Strains, plasmids and oligonucleotides used in this work are listed in Supplementary Tables S2, S3 and S4, respectively. YJE004 (BW25113/pET15K-NTT4/*nadD*::*cat*) NAD auxotrophic strain was a gift from Dr Zhao, Dalian National Laboratory for Clean Energy, China, Δ*hns*, Δ*nudC* were from Keio collection, Δ*rnc* strain AB301-105 from Yale *E. coli* stock centre, *rpoB* mutants S512F, Q513L, H526Q, R529H, I572F were spontaneous rifampicin resistant derivatives of WT strain, *rne* temperature-sensitive strain (*rne*-3071(ts)) was a gift from Prof Ben Luisi, University of Cambridge. Plasmids pET28*orn* (expressing *E. coli* Orn under IPTG inducible T7 promoter), pJW370 (pACYC184: *P_RNAI_ cI* (1–90) *lacZ*, pJW371 (pACYC184: *P_RNAI_* RNAI), pJW399 (pACYC184: *P_RNAI_* RNAI, antisense *P_trc_* RNAII (1–128) T7 terminator) were constructed in this work.

### Proteins

*Escherichia coli* RNA polymerase, from WT and spontaneous Rifampicin -resistant strain *rpoB R529H* were isolated as in (21), initiation factor σ^70^ and NudC were overexpressed and isolated as in (5,22,23). *orn* oligoribonuclease gene was cloned into expression vector pET28, N-terminal Hisx6-tagged protein was expressed and isolated as described in (5). RNase E protein was a gift from Prof Ben Luisi.

### Bacterial growth

*Escherichia coli* strain K-12 BW25113 was used as a wild type strain in this study. Cultures were grown at 37°C with shaking (200 rpm) in Luria-Bertani (LB) broth in aerobic conditions. For anaerobic growth, cultures were grown in a 60 ml syringe containing several sterile glass beads for more efficient shaking, with the same media and shaking conditions as aerobic growth. Novobiocin (Sigma-Aldrich; 25 μg/ml) was used to treat *E. coli* cultures for 5 min at 37°C with shaking before being harvested for analysis. NAD auxotrophic strain was grown in LB supplemented with 50 μM NAD overnight and then diluted 100 times into LB with the required concentration of NAD (1 μM–1 mM). Cells were harvested for RNA isolation and NAD/ATP quantification during the early stationary phase (OD_600_∼ 1.0), unless overwise indicated. To limit nitrogen levels, cells were grown in Gutnick minimal media (33.8 mM KH_2_PO_4_, 77.5 mM K_2_HPO_4_, 5.74 mM K_2_SO_4_, 0.41 mM MgSO_4_), supplemented with Ho-LE trace elements (24), 0.2% glucose and nitrogen source (3 mM NH_4_Cl). Inhibition of proton pump by AsO4^2-^ was done in LB medium supplemented with 10 mM sodium arsenate (NaAsO_4_) at OD_600_ = 1 for 15 min. Cultures were harvested by centrifugation at 8000g for 2 min to remove the medium. The cell pellet was flash frozen in liquid nitrogen and stored in −80°C until used.

### Determination of NAD(H) and ATP levels in *E. coli* cells

100 μl of cell culture were harvested by centrifugation at 9000g for 1 min at room temperature, the supernatant was decanted, and the cell pellet was flash frozen in liquid nitrogen and stored in −80°C until used. Total cellular NAD levels were quantified using the Sigma-Aldrich NAD/NADH Quantification Kit (MAK037). Free ATP was quantified with ATP bioluminescence assay kit, (Roche, cat. no. 11699709001), according to manufacturers’ instruction.

### RNA isolation and purification

Total RNA was extracted from *E. coli* cells grown at 37°C in LB medium until the OD_600_ specified in experiments. For strains containing plasmid pCA NudC, expression of NudC was induced with 0.5 mM isopropyl-β-d-thiogalactoside (IPTG) at OD_600_ = 0.8. Cells were harvested by centrifugation at 9000g for 1 min at room temperature, the supernatant was decanted. The cell pellet was then flash frozen in liquid nitrogen and stored in −80°C until used. For RNA extraction, the frozen cell pellet was resuspended in 1 ml of TRI reagent (Sigma Aldrich; T9424) per 3 OD units of bacterial culture (equal to number of cells in 3 ml of a culture of OD_600_ = 1) and lysed by incubation at 70°C for 10 min, following the manufacturer's instructions. The sample was then cooled on ice, and 200 μl of chloroform was added. The mixture was vortexed vigorously for 30 s and then centrifuged at 4°C, 20 000g for 10 min. The upper phase was extracted twice with the same volume of phenol/chloroform solution (5:1 ratio), and the RNA was precipitated with 2.5 M ammonium acetate and 3 volumes of ethanol in −20°C for 1 h. The RNA pellet was collected by centrifugation at 4°C, 20 000g for 30 min, washed with 70% cold ethanol, air dried for 10 min, resuspended in 50 μl of RNAse-free water and stored in −80°C until use. RNA quality and concentration were determined using a NanoDrop spectrophotometer.

### FluorCapQ quantification of NAD capping on total RNA

RNA for FluorCapQ was further purified to remove free NAD that would interfere with the analysis and bias the results. RNA was dissolved in 2 M urea and 10 mM Tris pH 7.6, heated for 2 min at 65°C, and purified using the Monarch® RNA Cleanup Kit (50 μg) (New England Biolabs) according to the adjusted manufacturer's instructions for the purification of RNA ≥ 15 nt. The RNA yield was quantified using a NanoDrop spectrophotometer. 10 μg of RNA was dissolved in 40 μl of RNase-free water and mixed with 15 μl of a 25 mM solution of 2-acetyl benzofuran (dissolved in ethanol) and 15 μl of 0.5 M KOH. The mixture was incubated on ice for 20 min, then 70 μl of formic acid was added and the mixture was incubated at room temperature for an additional 20 min. The fluorophore thus produced was found to be stable in the dark at room temperature for 2 h (25). Standard curve samples were prepared in the same way, just dilutions of NAD were used instead of total RNA. The resulting solution was transferred to a black 96-well microplate (Fluotrac, Greiner) and fluorescence was measured by BMG Clariostar fluorimeter (BMG Latec) (using excitation wavelength of 420 ± 40 nm, emission wavelength of 480 ± 40 nm). The NAD levels in each RNA sample were calculated by comparing the absorbance values to a standard curve generated using known concentrations of NAD.

### Northern blotting and boronate acrylamide electrophoresis for detection and quantitation of NAD-capped RNA *in vivo*

RNA samples were treated/untreated with 400 nM NudC in NudC buffer as specified, precipitated, resuspended in RNA loading dye and denatured (20 mM EDTA, 0.025% bromophenol blue, 0.025% xylene cyanol, 7 M Urea, 1× TBE, 100 mg/ml heparin, and 80% formamide at 95°C for 10 min). NCIN capping of abundant RNAs was analysed by a procedure consisting of: (i) electrophoresis on 7.5 M urea, 1× TBE, 10% polyacrylamide gels supplemented with 0.33% 3-acrylamidophenylboronic acid (APB; Sigma Aldrich); (ii) transfer of nucleic acids to a BrightStar™-Plus Positively Charged Nylon Membrane (Invitrogen) using a semidry transfer apparatus at 15 V for 1 h (Bio-Rad); (iii) immobilisation of nucleic acids by UV crosslinking (UV crosslinker (AH) at 1200 μJ/cm^2^); (iv) prehybridisation 30 min in 37°C in SES1 buffer (0.5M sodium phosphate buffer pH7.2, 7% SDS, 10 mM EDTA pH 8), (v) incubation with a ^32^P-labelled oligodeoxyribonucleotide probe complementary to the target RNAs (^32^P-labelled using T4 polynucleotide kinase and [γ ^32^P]-ATP [Perkin Elmer]) overnight in 37°C in SES1 buffer; (vi) washing 2 times 20 min with washing buffer (0.1% SDS, 1× SSC (Thermo Scientific) and (vii) storage-phosphor imaging (AmershamTyphoon). Bands corresponding to uncapped and NCIN-capped RNAs were quantified using ImageQuant software. The percentages of NAD^+^-capped RNA (5′-NAD) to total RNA were determined from three biological replicates.

### Detection of low abundant RNAs by northern blotting with RNA probe

Less abundant sRNAs/mRNAs were detected using radiolabelled RNA probes prepared by T7 *in vitro* transcription with α^32^P UTP (see the section ‘*In vitro* transcription with T7 RNAP’). Template for SibD probe was amplified from *E. coli* genome using primers JW253 and JW254 (Supplementary Tables S4). The RNA probe was purified using Micro Bio-Spin™ *P*-6 Gel Columns (Biorad), denatured 5 min in 95°C and cooled on ice. The membrane with immobilized RNA was prehybridized in buffer (1% SDS, 6X SSPE (Invitrogen), 10× Denhardt's Solution (Invitrogen),1M NaCl, sodium phosphate buffer pH 7.4, 50% formamide, yeast tRNA 200 mg/ml (Sigma Aldrich)) for 1h in 42°C and hybridized with the RNA probe overnight at 42°C. The membrane was washed two times with 2× SSC for 10 min, 2× with wash buffer (2× SSC, 1% SDS) at 65°C for 30 min and two times with 0.1× SSC at 65°C for 30 min. The other steps of the procedure were the same as described above.

### DNAzyme cleavage of RNA

Cellular RNAs longer than 200 nt (*cI-lacZ* fusion mRNA) were processed with DNAzyme before the electrophoresis step: 40 μg of total RNA was mixed with 1 mM DNAzyme (JW131) in buffer containing 10 mM Tris pH 8.0, 50 mM NaCl, 2 mM DTT (total volume 50 μl). Samples were heated to 85°C for 5 min and cooled to 37°C (1°C per 30 s). MgCl_2_ was added to a final concentration of 10 mM and, when present, NudC was added to 400 nM. Reactions were incubated for 60 min at 37°C, then 100 μl of DNAzyme stop solution (0.6 M Tris–HCl pH 8, 18 mM EDTA pH 8, 0.1 mg/ml glycogen) and 500 μl of ethanol were added. Samples were centrifuged (30 min, 21 000 g, 4°C), the supernatant removed, and the pellet resuspended in RNA loading dye.

### *In vitro* transcription with T7 RNA polymerase: NAD-RNA/ppp-RNA, size standards for Northern blots

Specific RNAs used in this study were generated by *in vitro* transcription using T7 RNAP. DNA templates containing class I T7 promoter (for transcripts starting with +1G) and class II T7 promoter (transcripts starting with +1A or NAD) were produced by PCR and purified by PCR purification kit (Qiagen) by manufacturer's instructions.

To generate size standards for Northern blot analysis, we produced RNAs using T7 RNA polymerase with a class II promoter. Two types of RNA were generated: triphosphorylated RNA and NAD-capped RNA. The RNA synthesis reactions were carried out in T7 transcription buffer containing 40 mM Tris–HCl (pH 7.9), 10 mM MgCl_2_ and 5 mM DTT. For the synthesis of triphosphorylated RNA, the reaction mixture contained 1mM each of ATP, CTP, GTP and UTP. For the synthesis of NAD-capped RNA, the reaction mixture contained 1 mM each of CTP, GTP, and UTP, 0.2 mM ATP and 4 mM NAD. Usually 6 pmol of T7 RNAP was used in 100 μl reaction volume. After incubating the reactions at 37°C for 2 h, the RNA was purified using Micro Bio-Spin™ *P*-6 Gel Columns (Biorad) according to the manufacturer's instructions. When preparing radiolabelled RNA, UTP was reduced to 0.1 mM and complemented with 1 μl of α^32^P UTP (Perkin Elmer). The purified RNA was then analysed on a denaturing polyacrylamide gel to confirm the expected size of the RNA products.

### *In vitro* transcription by *E. coli* RNAP

A total of 0.3 pmol of wild-type or mutant *E. coli* RNAP core with 1 pmols of σ^70^ and 2 pmol of linear DNA fragment containing p_RNAI promoter were incubated at 37°C for 10 min in 10 μl of transcription buffer (20 mM Tris–HCl (pH 7.9), 40 mM KCl, 0.1 mM ethylenediaminetetraacetic acid) at 37°C. For experiments on panel 3D plasmid template pCDF1b was used either supercoiled or linearized by restriction with EcoRI endonuclease. Then nucleotides or nucleotides analogues were added to the final concentration of 500 μM (unless otherwise indicated). For competition assays NAD concentration was kept constant at 250 μM and ATP concentrations ranging from 20 to 1000 μM, as shown on corresponding Figures were used. Transcription was initiated by the addition of 10 mM MgCl_2_, 50 μM (α^32^P)-CTP, 12.5 Ci/mmol (Hartmann Analytic). Reactions were stopped after 5 min incubation at 37°C by the addition of formamide-containing loading buffer. Products were separated on denaturing polyacrylamide gels (30% acrylamide, 3% bis-acrylamide, 6 M urea, 0.5× Tris–borate EDTA buffer), revealed by PhosphorImaging (GE Healthcare), and analysed using ImageQuant software (GE Healthcare). For apparent *K*_M_ determination, NTPs and analogues were used in concentrations ranging from 10 μM to 1 mM and constant 50 μM CTP concentration. The band intensities were quantified using ImageQuant software; to calculate the initial reaction rate these numbers were divided by reaction duration time. These data were fitted to hyperbolic equation: *f* = *y*_0_ + a*x/(*b* + *x*) using non-linear regression algorithm in SigmaPlot software. Proportion of NAD-RNA in competition experiments was calculated as a percentage from total signal from all products in the corresponding lane.

### RNAI-RNAII binding assay

The assay was done as described before (26,27) with few adjustments. Briefly: radiolabelled RNAI species (NAD-RNAI, ppp-RNAI, NAD-RNAI-A_6_, ppp-RNAI-A_6_) were *in vitro* transcribed, analysed and quantified by PAA gel electrophoresis and radiography, to use the same amount of each RNA per reaction.

RNAII was DNAsed, purified by Monarch® RNA Cleanup Kit (New England Biolabs) and quantified by nanodrop. All RNAs were denatured in water (85°C for 2 min, cooled on ice). An equal mass of radiolabelled RNAI was mixed with 2× serial dilution of RNAII (32, 16, 8, 4, 2 and 0 ng) in binding buffer (20 mM Tris–HCl pH 7.6, 100 mM NaCl, 10 mM MgCl_2_). The final volume of the reaction was 5 μl and it was kept at 37°C for 8 min. For the kinetics experiment in Supplementary Figure S4D 200 nM of RNAII was used, and incubated for the 30′’, 1′, 5′, 10′, 15′ and 20′.The reaction was stopped with 10 μl of gel loading buffer (7M urea, 20 mM Tris–HCl pH 7.6, 0.5% SDS, 10 mM EDTA, 0.005% bromophenol blue, 0.01% xylene cyanole) and 3 μl of each sample were loaded without heating on 10% polyacrylamide gel containing 8 M urea in 1× TBE buffer. The electrophoresis was run in a pre-cooled 1× TBE buffer in a cold room at 8°C at constant power of 10 W.

### RelE-printing/binding of 70S ribosome to leaderless RNA, RelE assay

Purification of components for ribosome binding to mRNA and RelE cleavage was done as described previously: 70S ribosomes (28), RelE (29) (plasmids kindly provided by Kenn Gerdes, Copenhagen University). tRNA^fMet^ was isolated by an adjusted method developed by Yokogawa *et al.* (30) using a complementary biotinylated probe JW32 (Supplementary Tables S4) and streptavidin agarose (Invitrogen). Briefly: streptavidin agarose was washed 3 times in 500 μl of hybridisation buffer (10 mM Tris–HCl (pH 7.6), 0.9 M NaCl, 0.1 mM EDTA). 200 μl of biotinylated oligonucleotide JW32 (100 mM) was mixed with 100 μl of washed streptavidin agarose and 5 μg of tRNA from *E. coli* MRE 600 (Roche) in 1 ml of hybridisation buffer. The mixture was heated to 70°C for 10 min and cooled slowly to room temperature while agitating. Agarose was washed 4 times with 400 μl of washing buffer (10 mM Tris–HCl, pH 7.6). The captured tRNA^fMet^ was then eluted twice by heating the beads to 70°C for 5 min in 100 μl of wash buffer, fast spinning, and taking the supernatant. RNA was precipitated and resuspended in nuclease-free water.

Model leaderless mRNA (70 nt long 5′-portion of *cI* mRNA of phage lambda) containing 5′-ppp or 5′-NAD was synthesized by T7 RNAP using a template containing class II T7 promoter as described in section for *in vitro* transcription with T7 RNA polymerase. The transcription template was amplified using oligo JW100 as a template and JW1, JW2 as PCR primers. mRNA was radiolabelled at the 3′-end using T4 RNA ligase I (NEB) and [5′-^32^P] pCp (Perkin Elmer) according to the manufacturer's instructions just the reaction was performed for 1 h at room temperature. The RNA was purified by Monarch® RNA Cleanup Kit (New England Biolabs). Binding of ribosomes to leaderless mRNA was tested by assembling 10–500 fmol of 70S ribosomes with 10 fmol of NAD/ ppp-leaderless mRNA in translation buffer (10 mM Tris–HCl pH 7.4, 60 mM NH_4_Cl, 10 mM Mg(OAc)_2_, 6 mM β-mercaptoethanol) in the presence or absence of 10 pmol of tRNA^fMet^. The mixture was incubated at 37°C for 10 min. When indicated, 12 pmol of RelE was added and incubated at 37°C for an additional 10 min. The reaction was stopped by adding 10 μl of transcription loading dye and run on 15% polyacrylamide gel, dried, exposed to storage phosphor screens, scanned and visualized by Typhoon, Cytiva and quantified by ImageQuant software.

### Affinity kinetics measured by Octet® RED96e

The components used for RelE toeprinting assay were used for measurements of affinity constants by Octet® RED96e using Bio-Layer Interferometry (BLI) based on fiber-optic biosensors.

200 nM model leaderless mRNA (70 nt *cI* mRNA; see Toeprint experiment with RelE toxin) was hybridized to biotinylated DNA oligo (JW10; 100 nM) in annealing buffer (10 mM Tris–HCl pH 6.8, 50 mM NaCl) by heating to 65°C for 5 min and cooling 1°C per 30 s to room temperature. The streptavidin sensor of Octet® RED96e was equilibrated in an annealing buffer and the RNA-DNA hybrid was loaded on the sensor. The sensor was blocked with biotinylated protein A to prevent unspecific interactions and washed with translation buffer (10 mM Tris–HCl pH 7.4, 60 mM NH_4_Cl, 10 mM MgOAc). 202 and 36 nM 70S ribosomes were mixed with 2-fold molar excess of tRNA^fMet^ in translation buffer and loaded to the sensor with the attached DNA-RNA hybrid. The binding and dissociation of 70S ribosomes were monitored and affinity constants were calculated.

### β-Galactosidase assay of proteins coded by leaderless mRNA

A short initial portion of phage λ *cI* gene (10 N-terminal amino acid residues) was fused with *lacZ* gene and cloned into pACY184 under RNAI promoter resulting in the plasmid JW370. This construct produced a leaderless mRNA coding *cI-lacZ* fusion protein and was transformed into *E. coli* WT and Δ*nudC* strains. These strains were grown in LB medium until the exponential phase and then treated with kasugamycin (final concentration of 750 μg/ml) for 30 min. β-Galactosidase activity of CI-β-gal fusion was measured as described (31).

### Preparation of RNA template for *in vitro* translation

The RNA template for translation (leadered or leaderless phage lambda *cI-lacZ* mRNA) was prepared by T7 RNAP dependent *in vitro* transcription using dsDNA as a template. dsDNA templates were amplified by PCR using the plasmid JW195 and oligos W114, W115 as primers for the leadered template and the plasmid JW190 and oligos JW115, JW116 for the leaderless template. The resulting dsDNA products contain class II T7 promoter followed by AUG codon (leaderless) or with leader sequence from pCDF expression vector (leadered) and a coding sequence of 714 nt (coding for 10 N-terminal amino acids of phage lambda CI protein fused to N terminal part of LacZ). The mRNAs were produced in two forms: with 5′-ppp and 5′-NAD as described in the Methods′ section ‘*In vitro* transcription with T7 RNA polymerase’. mRNA was treated with DNAse I (New England Biolabs) and purified by Monarch® RNA Cleanup Kit (New England Biolabs).

To check the quality and quantity of the mRNA, it was labelled by [5′-^32^P] pCp (Perkin Elmer), resolved on 5% PAA gel containing 0.33% APB and the respective bands were quantified to assure that the same amount of RNA will be used in *in vitro* translation assay.

### *In vitro* translation

*In vitro* translation experiment was performed using *E. coli* S30 Extract System for Linear Templates (Promega), according to the manufacturer's instructions for radiolabelled translation. The reaction was supplemented with [^35^S]-Methionine (Perkin Elmer) and 2.4 μg of each mRNA template per sample and run for 2 h at 37°C. The translated protein (2 μl) was mixed with loading buffer, denatured and analysed on NuPAGE™ 4 to 12%, Bis–Tris SDS PAGE gel (Invitrogen). Besides, the Novex™ Sharp Pre-stained Protein Standard (Invitrogen) was run on the gel. After the run, each of the pre-stained bands of the standard was labelled with 0.1 μl of diluted [^35^S]-methionine by piercing the gel. The gel was dried, exposed to a storage phosphor screen, and scanned by Amersham Typhoon.

### *In vivo* antisense RNA protection of capping

RNAI from the plasmid pCDF-1b (colE1 origin of replication) under its native promoter was cloned into the plasmid pACYC184 (p15A origin of replication) using Gibson assembly (New England Biolabs) generating the plasmid JW371, this plasmid was transformed into wt and Δ*nudC*, *E. coli* strains. To express the antisense RNA to RNAI (5′-end of RNAII), we cloned a strong *P_trc_* promoter upstream of the 5′-end of RNAII and T7 terminator (efficiently recognized by *E.coli* RNAP (32)) downstream of RNAII fragment. The whole RNAI and 5′-end of RNAII complementary to RNAI is transcribed from the resulting plasmid (JW399). Since RNAII is not full-length, it does not promote replication of the plasmid and it is not a functional origin of replication (33).

Bacterial strains were grown in LB medium and harvested for RNA extraction at exponential (OD_600_= 1) and stationary (OD_600_= 3) phases. RNA was isolated as described. NAD capping of RNAI was assessed by Northern blotting of total RNA isolated from these strains and separated in 10% PAA gel containing 0.33% APB as described. RNAI was detected by Northern blotting by a DNA probe (JW198) labelled with T4 polynucleotide kinase (New England Biolabs).

### Processing of NAD-RNA *in vitro* by RNaseE and olgoribonuclease of *E. coli*

To determine the susceptibility of NAD-capped and triphosphorylated RNA *in vitro*, equal amounts of radiolabelled RNAI were incubated with or without 50 μM RNAse E at 37°C for various time (0, 5, 10, 15, 20 and 30 min) in reaction buffer (50 mM Tris–HCl, pH 7.5, 50 mM KCl, 5 mM MgCl_2_). At each time point, the reaction was stopped by adding an equal volume of transcription loading dye. The samples were denatured (95°C, 2 min) and separated by 10% PAA containing 0.33% APB as described.

Dinucleotide substrates for oligoribonuclease (Orn) were prepared in *in vitro* transcription reaction on linear PCR-generated template containing promoter *p_RNAImod* (5) with 500 μM ATP or NAD and 25 μM [α-^32^P]CTP (Perkin Elmer) using hexa-histidine tagged *E. coli* RNA polymerase. After incubation in reaction buffer (50 mM Tris–HCl, pH 7.5, 50 mM KCl, 5 mM MgCl_2_) for 15 min at 37°C RNA polymerase was removed by addition of Ni-NTA agarose beads and spinning down the suspension. Supernatant containing dinucleotides (ppp-A-p-C or NAD-p-C) was transferred to a new tube where 100 nM Orn was added and incubated for further 10 min at 37°C. The reaction was stopped by adding equal volume of transcription loading dye. The samples were denatured (95°C, 2 min), separated by 33% PAAG and visualized by radiography.

## Results

### FluorCapQ, a new sensitive NAD-RNA quantification method

**Figure 1.**
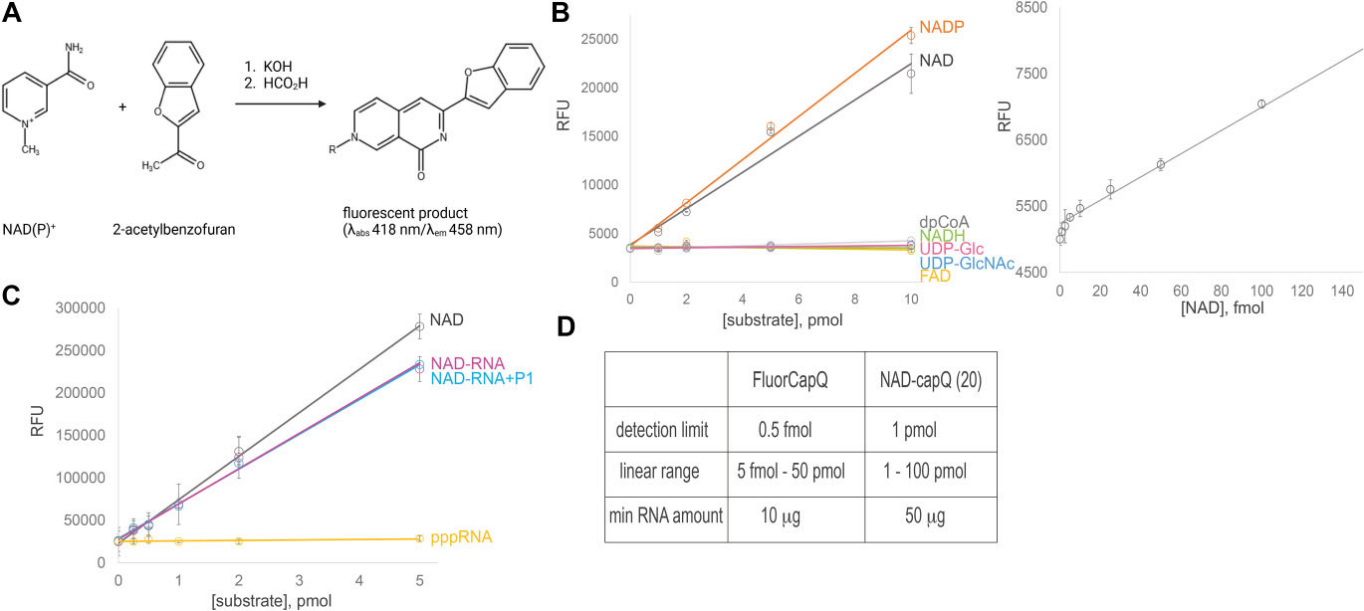
FluorCapQ method for quantification of NAD-RNA. (**A**) A scheme of FluorcapQ protocol. A sample containing NAD(P)+ is mixed with 2-acetylbenzofuran and incubated with potassium hydroxide. Afterwards it is heated in formic acid to produce a fluorescent compound. (**B**) Various amounts of cofactors (NAD^+^, NADH, FAD, NADP+, dpCoA, UDP glucose, UDP GlcNAc) were assayed with FluorCapQ method and the resulting fluorescence was measured. The plot on the right shows the curve for low range of NAD concentrations. Data were fit to *y* = *y*_0_+ *ax* equation. (**C**) Equimolar amounts of free NAD or NAD-RNA and ppp-RNA were subjected to FluorCapQ. NAD-RNA was or was not treated by nuclease P1 prior to FluorCapQ. The relative fluorescence was measured. Data were fit to *y* = *y*_0_+ *ax* equation. (**D**) Comparison of detection limits and linear ranges of FluorCapQ versus NAD-capQ method (Putt *et al.* (25), NAD/NADH Quantification Kit, Sigma Aldrich, MAK037).

To answer questions related to the physiological context of NADylation of RNA, we needed to relate intracellular ATP and NAD concentrations in relevant conditions (measured by commercial kits) to the amount of NAD being covalently attached to RNA. For the quantification of the bulk RNA NADylation, we developed a new fluorescent method, FluorCapQ. This sensitive, simple and reproducible method is a modification of an assay developed by Moriya et al., (34) based on the properties of *N*-alkylpyridinium compounds (in this case nicotinamide moiety of NAD), which convert to fluorescent molecules through reaction with a ketone (e.g. acetophenone or 2-acetylbenzofuran) followed by heating in excess acid (25,34) (Figure 1A). The resulting fluorescent compound can be quantified. FluorCapQ does not detect other cofactors known to be attached to RNA. Out of the tested cofactors, only NAD^+^ and NADP^+^ (not NADH, FAD, dpCoA, UDP glucose or UDP GlcNAc) generated the fluorescent signal (Figure 1B). NADP^+^ is not efficiently incorporated into RNA by bacterial RNAP (5). However, we cannot exclude that NAD capped RNA can be phosphorylated by some of the abundant cellular kinases to form NADP^+^. Quantification of NAD attached to RNA does not require nuclease treatment (Figure 1C). The sensitivity and linear range of the FluorCapQ are superior compared to the previously reported colorimetric NAD-CapQ method (13) (Figure 1D). Importantly, the concentrations of NAD-RNA measured by FluorCapQ method closely match those measured by LC-MS (7 versus 2.5 fmol NAD/μg RNA, respectively (2,8). For reliable quantification of NAD capping, proper removal of free NAD is crucial as discussed in the Supplementary information and Supplementary Figure S1.

### ATP absolute concentration and [ATP]/[NAD] ratio determines efficiency of NAD incorporation into RNA

The proposed mechanism of *de novo* NAD incorporation into RNA is based on the ability of RNAP to use it as a non-canonical initiating substrate. The extent of RNA NADylation potentially depends on two parameters – absolute NAD concentration and its ratio to a canonical RNAP substrate, ATP.

We found that in the most widely used laboratory growth conditions for *E. coli*, i.e. during aerobic growth in LB medium with shaking in a flask, intracellular NAD concentration is about ∼250 μM in the late exponential phase. This concentration remains relatively stable through the stationary growth phase until OD_600_∼4.5 (Supplementary Figure S2). Similarly, ATP levels do not vary in the range from OD_600_ 0.5 to 4.8, remaining at ∼1mM (Supplementary Figure S2), which is in agreement with previously published results (35).

**Figure 2.**
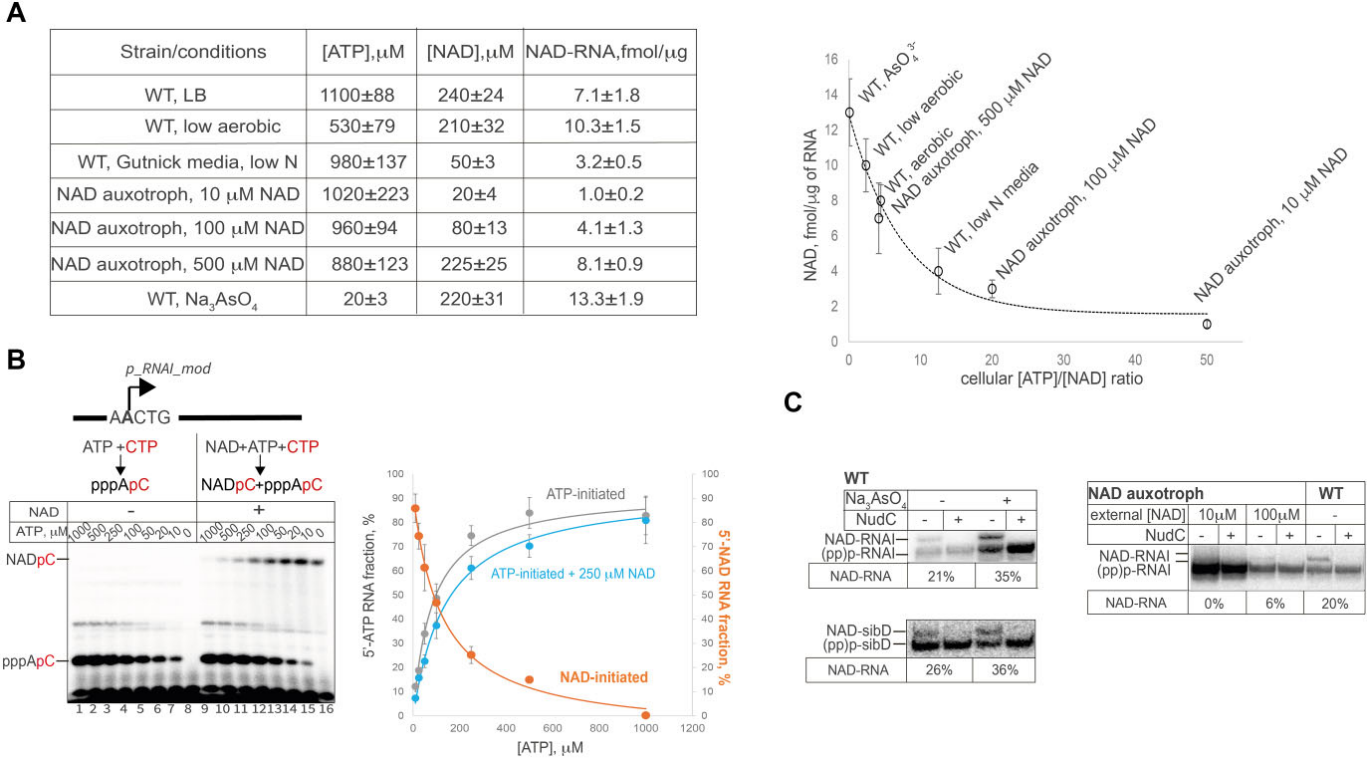
[ATP]/[NAD] ratio determines cellular RNA NADylation. (**A**) RNA 5′-NADylation is a function of [ATP]/[NAD] ratio. The table shows NAD content on total RNA isolated from cells at indicated conditions; specified in Materials and methods, *n*, the number of biological replicates = 3. [ATP] and [NAD] concentrations were measured in parallel; *n* = 3. Right, the plot of NADylated RNA concentration dependency on intracellular [ATP]/[NAD] ratio. Data were fit to *y* = *y*_0_+(a*b)/(*b* + *x*) equation. (**B**) *In vitro* competition assay for NAD vs ATP as initiating substrate in dinucleotide synthesis by WT RNAP in the presence of [α-^32^P]-CTP on *p_RNA_mod* promoter. Scheme of the reaction substrates and products is shown above the representative gel image. Assay was done with increasing concentrations of ATP in the absence or the presence of the 250 μM NAD. For the plot on the right, data were fit to *y* = *y*_0_+ (a*b)/(*b* + *x*) equation. Values are mean ± SD from three independent experiments. Percentage reflect the fraction of the product initiated with indicated substrate, from total signal intensity of all transcription products in the lane imaged by Phosphor Imager and quantified by ImageQuant TL software. (**C**) [NAD]/[ATP] ratio determines efficiency of RNAI and sibD NADylation *in vivo*. Representative northern blots for RNAI and SibD RNAs are shown (images of the full blots are shown on Supplementary Figure S3). Percentage of NAD-RNA is shown below the gels, as measured by Northern blot of total RNA, either untreated or treated with 20 μM NudC with DNA probe against RNAI or RNA probe against *sibD*. Total RNA was separated on denaturing PAAG supplemented with 0.33% 3-acrylamidophenylboronic acid. Experiments were performed in three biological replicates.

To control NAD concentration in the cell, we have employed the NAD auxotrophic strain of *E. coli*, YJE004, where levels of intracellular NAD can be manipulated by varying concentrations of NAD in growth media (36). Using this strain, we tested levels of extracellular NAD from 10 μM to 0.5 mM, which translated into intracellular NAD concentrations from 20 μM to 220 μM (Figure 2A). As an alternative way to lower intracellular NAD concentration, we used WT strain grown in Gutnick media with limited nitrogen concentration.

To investigate the effect of NAD competition with ATP, we lowered down [ATP] by: (i) placing cells in microaerobic conditions, which has led to a twofold decrease of [ATP] to ∼500 μM, or (ii) treating cells with AsO_4_^3−^ (inhibitor of ATP synthase (37)), which has led to a significant drop in the concentration of ATP to ∼20 μM. [NAD] in these conditions remained unchanged, ∼220 μM (Figure 2A, left panel).

We have found that in general, steady-state levels of NAD-RNA had followed a simple trend, correlating with [ATP]/[NAD] ratio, from 1 fmol NAD/1 μg of total RNA in YJE004 ([ATP]/[NAD] ≈ 51) to 12.5 fmol NAD/μg of total RNA ([ATP]/[NAD] ≈ 0.09) in cells treated with arsenate (Figure 2A, right panel). A similar trend was observed in *in vitro* competition experiment where the efficiency of dinucleotide RNA formation was assessed at a physiological concentration of NAD, 250 μM supplied with increasing ATP concentrations (Figure 2B, orange curve on the plot).

In parallel, to corroborate *in vivo* bulk measurements, we assessed NADylation specifically of RNAI and SibD, two highly NADylated RNAs of *E. coli* (6), by Northern blots of total RNA samples isolated from given strains and conditions. These samples were run on denaturing polyacrylamide gels containing 3-acrylamidophenylboronic acid, allowing separation of 5′-NAD and 5′-ppp RNA species (38,39). Consistently with bulk RNA NADylation measurements, the NAD-RNAI content grew from 21% to 35%, NAD-SibD from 26% to 36% in cells grown in LB compared with cells treated with AsO_4_^3−^ (Figure 2C, Supplementary Figure S3A, B). Using NAD auxotrophic YJE004 strain, we demonstrated that levels of 5′-NAD RNAI are negligible at cellular [NAD] below ∼100 μM (Figure 2C, Supplementary Figure S3C).

### Initiating substrate coordination in RNAP active site, genomic DNA accessibility and supercoiling regulate 5′-NADylation of RNA

**Figure 3.**
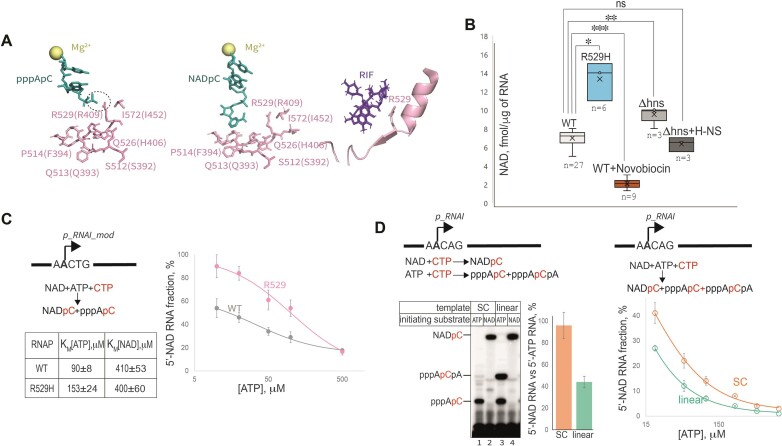
RNAP Rif pocket residue R529 and DNA supercoiling influence RNA NADylation. (**A**) Residue R529 of β subunit coordinates triphosphate moiety of pppApC short RNA product in the active site of RNAP. Structures of *Thermus aquaticus* RNAP (residues numbers in brackets, and corresponding *E. coli* RNAP residues numbers without) open promoter complex with pppApC RNA, PDB: 5D4C and NADpC, PDB: 5D4D and structure of *E. coli* RNAP with Rifampicin, PDB: 4KMU. Mg^2+^ is shown in beige, fragment of β subunit with R529 is in pink, RNA is in blue/turquoise, Rifampicin in purple. (**B**) R529H mutation in β subunit, changes in DNA supercoiling and H-NS deletion affect NADylation of RNA. NAD-RNA concentration in total RNA isolated from WT in the presence or absence of novobiocin, and mutant strains was measured with FluorCapQ, *n* = number of independent biological replicates. *P*-values (two-sided Student's *t*-test) are shown above the histogram wit asterisks, * signifies *P*-value ≤ 0.05, **P-value≤ 0.01, ***P-value ≤ 0.001. (**C**) R529H RNAP is more efficient in NAD incorporation. *In vitro* ATP/NAD competition initiation experiment was done as in Figure 2B for WT and R529H mutant RNAPs, except NAD concentration was kept at 250 μM. Scheme of experiment is shown on the left of the plot, table below shows values of apparent Michaelis constants (*K*_M_s) for WT and R529H mutant, values are mean ± SD from three independent experiments. Competition experiments data are plotted as mean values ± SD as error bars from three independent biological experiments. (**D**) *In vitro* transcription initiation with NAD on supercoiled plasmid template is more efficient compared to linearized template. Left, the efficiency of initiation was assessed in separate experiments, using either ATP or NAD as initiating substrate, on supercoiled (SC) and linear plasmid template (gel image and bar plot on the left). Please note that on plasmid template the initial transcribed sequence allows RNAP to make di- and trinucleotide RNAs in reaction containing ATP and [α-^32^P]-CTP, but only dinucleotide in reaction with NAD and [α-^32^P]-CTP. Right: the linear plot shows results of the competition experiment using supercoiled and linear templates as in B. Data were fit to *y* = *y*_0_+(a*b)/(*b* + *x*) equation. Values are mean ± SD from three independent experiments.

The existence of RNA 5′-NADylation implies that RNAP active site incorporates initiating substrate with low accuracy. Could RNAP active site be modified to further increase the efficiency of NAD incorporation? During/after synthesis of 2-nt long RNA, nicotinamide moiety of NADylated RNA makes different contacts with amino acid residues of the RNAP active site compared to the triphosphate of the triphosphorylated RNA, Figure 3A (6). Mutating residues in positions 513 and 516, located in Rifampicin pocket, were shown to decrease NAD incorporation into RNA *in vitro* (5). We tested mutant strains with substitutions in *rpoB* gene leading to amino acid changes in Rifampicin pocket—S512F, Q513L, P514C, H526Q, R529H, I572F (40,41), in FluorCapQ assay. Only R529H mutation caused an increase in NAD-RNA concentration (Figure 3B and Supplementary Table S1). In the structure of promoter open complex with dinucleotide substrate, R529 of β subunit coordinates triphosphate part of the 5′-ATP, therefore we suggest that arginine change to histidine decreased ability of RNAP active site to coordinate triphosphate moiety of ATP, thus creating preference for NAD incorporation (Figure 3A). *In vitro* mutant R529H RNAP was more efficient to incorporate NAD during transcription on *p_RNAI_mod* promoter in a competition experiment where reactions for dinucleotide synthesis were initiated with mixtures of 500 μM NAD and ATP at various concentrations (Figure 3C). These results agree with higher value of apparent *K*_M_ for ATP for R529H RNAP (table in Figure 3C), measured separately in *in vitro* dinucleotide synthesis experiments on *p_RNAI_mod* promoter.

Initiation of transcription is long known to be sensitive to DNA supercoiling (42–44). Could there be a connection between DNA supercoiling density and RNA NADylation? To test this hypothesis, we briefly treated cells with novobiocin, an inhibitor of DNA gyrase, which leads to a decrease in negative DNA supercoiling and found that NADylated RNA content was lowered down (Figure 3B). This result suggests that genomic DNA supercoiling could be one of the general factors controlling incorporation of NAD, and probably promoters prone to NADylation are those more sensitive to supercoiling. These results were supported by *in vitro* test where on *p_RNAI* promoter on a supercoiled plasmid pCDF-1b template the efficiency of NAD incorporation was higher relative to ATP than on the same template linearized by restriction endonuclease cleavage (Figure 3D). Moreover, on a supercoiled template NAD was able to compete more efficiently with ATP, compared with linear template, as can be seen from the plot on the right side of Figure 3D and Supplementary Figure S4A. To simplify the analysis of NAD and ATP competition, we have changed A at position +3 of the *p_RNAI* promoter to T, calling resulting plasmid pCDF-1b_+3T. On this promoter NAD was also able to compete more efficiently with ATP as initiating substrate on supercoiled plasmid (Supplementary Figure S4B).

To further support this hypothesis, we tested levels of RNA NADylation in H-NS deletion strain. H-NS is a major nucleoid-organizing protein of *E. coli*, condensing DNA into the nucleoid. Deletion of H-NS increases the negative supercoiling of chromosomal and plasmid DNA (45). We found that deletion of H-NS led to an increase in overall RNA NADylation, while overexpression of H-NS from a plasmid led to a slight decrease in NADylated RNA concentration (Figure 3B). These results agree with the results of the experiment with novobiocin. However, we cannot rule out the alternative explanation - that at least some NADylated transcripts originate not from conventional promoters but as products of spurious transcription - removal of H-NS-dependent silencing of spurious transcription is known to activate some cryptic initiation sites (46,47). Consistently with this hypothesis, expression of initiation factor sigma D with G424D mutation (in a strain *ΔrpoD* p*RpoD^G424D^*), which is more ‘stringent’ and was shown to prevent spurious transcription initiated within a gene, decreased proportion of NADylated RNA (Supplementary Figure S4C).

### Functional binding of antisense RNAII is not affected by 5′-NAD of RNAI

**Figure 4.**
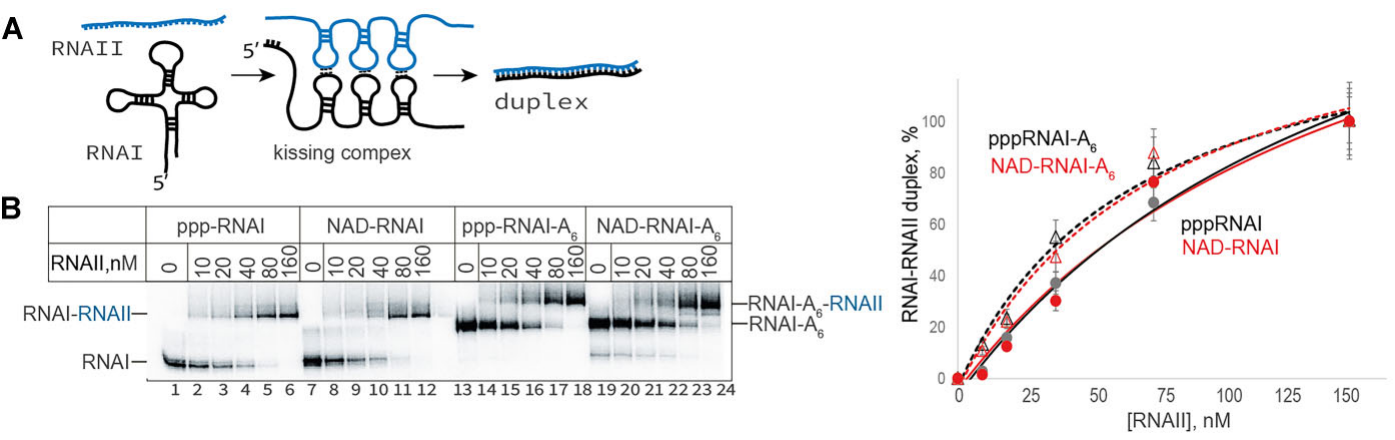
NADylated RNAI forms a duplex with RNAII with the same efficiency as triphosphorylated RNAI. (**A**) Scheme of the RNAI-RNAII duplex formation. (**B**) Duplex formation is not affected by RNAI NADylation. Internally labelled ppp-RNAI/NAD-RNAI (non-adenylated or hexa-adenylated at the 3′ end) was incubated with increased concentrations of unlabelled RNAII, the RNAI-RNAII duplex formation is observed as the retardation of RNAI mobility (upper band). Right: the percentage of RNAI in a duplex is plotted against RNAII concentration; triangles and dashed lines- non-polyadenylated RNAI in complex with RNAII, circles and full lines- polyadenylated RNAI in complex with RNAII. Complexes containing triphosphorylated RNAI are in black, and complexes with NAD-capped RNAI are in red. Values are mean ± SD from four independent experiments.

Since a majority of NADylated RNA are sRNA species, whose regulatory mechanism involves base-pairing with a target RNA, we tested if such base-pairing can be affected by 5′-NADylation. As a model, we used RNAI, which forms a full-length duplex with an antisense RNAII to control replication mechanism of ColE1-origin plasmid. Initially, RNAI forms a ‘kissing complex’ with RNAII, then this complex is stabilized by the 5′-end of RNAI interaction leading to the formation of a full-length duplex (Figure 4A) (48).

To test if 5′-NAD affects RNAI duplex formation with RNAII, we analysed efficiency of RNA-RNA duplex formation between *in vitro* synthesized RNAI and 128 nt long 5′-end complementary part of RNAII. RNAI was radioactively labelled, and the formation of RNAI-RNAII duplex, which migrates slower on PAAG under our experimental conditions, can be observed as an appearance of an additional band above the RNAI band.

Titrating RNAII, we have found that its affinity to ppp-RNAI is the same as to NAD-RNAI (Figure 4B). The main cellular form of RNAI is 3′ hexa-adenylated RNAI, which has a lower affinity to RNAII *in vivo* (27). Taking this into account, we also tried *in vitro* synthesized hexa-adenylated RNAI in the same experimental setup. Indeed, we found that affinities of adenylated form of RNAI was lower than non-adenylated, yet ppp-RNAI-A6 and NAD-RNAI-A6 affinities to RNAII were similar (Figure 4B). Taking saturating concentration of RNAII, we also showed that there is no significant difference between NAD-RNAI and ppp-RNAI kinetics of binding to RNAII (Supplementary Figure S4D).

### 5′-NADylated leaderless λ*cI*-based mRNA is bound by 70S ribosomes and efficiently translated

**Figure 5.**
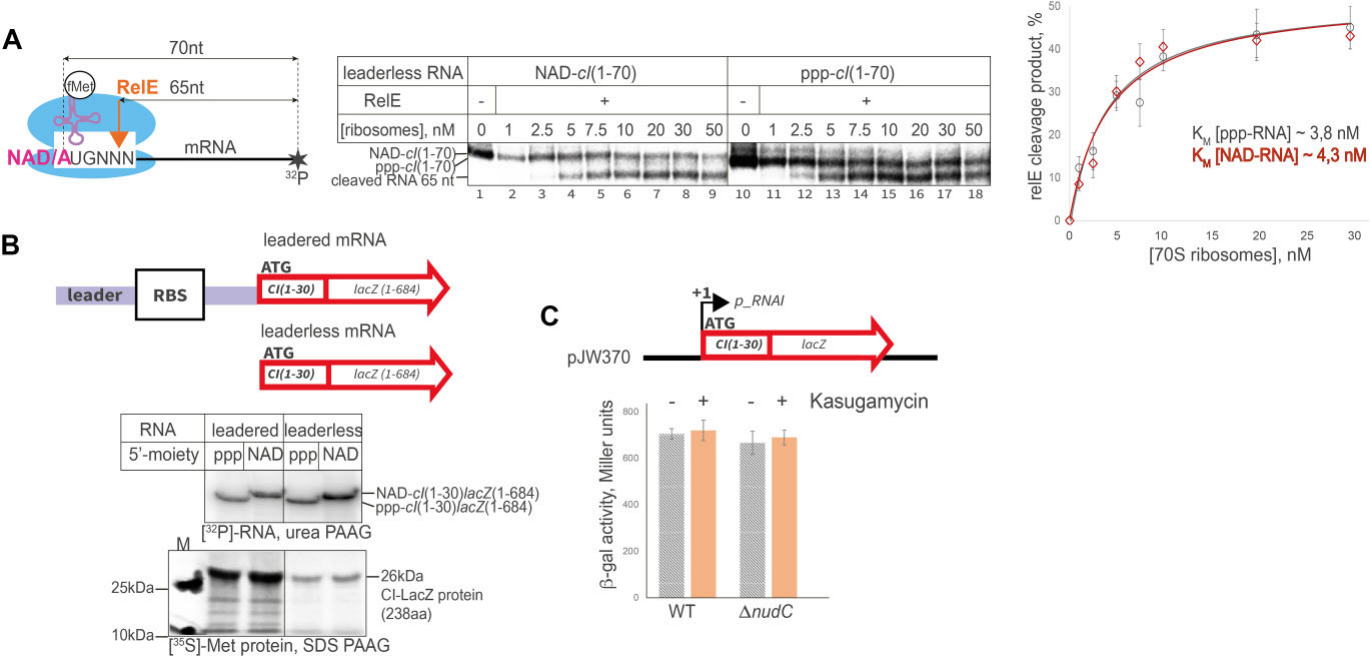
Translation of leaderless mRNA is not affected by 5′-NAD on RNA. (**A**) RelE cleavage of NAD- and ppp- mRNAs at increasing concentration of ribosomes. Left: scheme of the complex, leaderless mRNA starts directly with AUG/NAD-UG codon, middle: representative gel showing positions of original and RelE-cleaved mRNAs, produced *in vitro* by assembling translation initiation complex, including either leaderless 5′ ppp-mRNA or 5′ NAD-mRNA with indicated increasing concentration of 70S ribosomes, tRNA^fMet^ and RelE toxin where indicated. Right: plot of percentage of RelE-cleaved RNA vs. ribosome concentration. Values are mean ± SD from three independent experiments. (**B**) *In vitro* translation of leadered and leaderless NADylated or triphosphorylated mRNA. Top, a scheme of leadered versus leaderless mRNAs; RBS = ribosome binding site. Top gel panel –mRNAs labelled with [α-^32^P] UTP run on denatured urea gel, bottom: SDS-PAGE of the corresponding 26 kDa CI-LacZ fusion protein labelled with [^35^S] Met, produced using *E. coli* S30 Extract *in vitro* translation system, Promega. (**C**) β-Galactosidase activity of cell lysates expressing leaderless *cI-lacZ* fusion mRNA constructs in either WT or Δ*nudC s*trains with and without Kasugamycin, the specific inhibitor of leadered translation. Values are mean ± SD from three biological replicates.

Classic m^7^G capping is essential for the efficient translation of eukaryotic mRNA (49). NAD capping in eukaryotes appears to inhibit translation initiation (15), although this inhibition could be an indirect effect of preferential degradation of the 5′-NAD-RNA in the cell. Prokaryotic translation machinery has not evolved an apparent means for specific 5′-end of mRNA recognition. However, leaderless mRNA may represent a special case, where ribosome binds at the very 5′-end of mRNA, to the start codon. NADylation changes start codon from AUG to NAD-UG, Figure 5A. *E. coli* codes for only a few leaderless mRNAs. The best-studied model of leaderless translation is mRNA coding for CI repressor protein of bacteriophage λ (50).

Using *in vitro* produced 70-nt long 5′-fragment of *cI* mRNA, with either AUG (ppp-*cI*(1–70)) or NAD-UG start codon (NAD-*cI*(1–70)), we found that the complexes of these mRNAs with ribosomes and initiating tRNA^fMet^ have the same dissociation constants, ∼3.5 nM versus ∼4.3 nM, respectively, as measured by Bio-Layer interferometry (Octet instrument) using mRNA immobilized via annealing to biotinylated DNA oligo on a solid support. Independently, the position of the ribosome and its affinity was tested in a RelE-printing experiment (51), which uses RelE toxin to specifically cleave mRNA between the second and third nucleotides of the codon in the vacant A-site of the post-translocated ribosome (29) (scheme on Figure 5A). For this experiment we used the same ppp-*cI*(1–70) or NAD-*cI*(1–70) fragments which were radiolabelled at the 3′-end. Ribosome placement was not changed by 5′-NAD, judged by the same length of the RelE cleavage fragment. Affinities to mRNAs measured by titration of ribosomes followed by RelE cleavage were similar for ppp-*cI*(1–70) and NAD-*cI*(1–70), 3.8 nM versus 4.3 nM, respectively. These values correlated well with *K*_d_ values for the two mRNAs, measured with interferometry.

To assess the effect of 5′-NAD on the overall efficiency of translation of leaderless mRNA, 5′-NAD-mRNA and ppp-mRNA were produced, schemes on Figure 5B (leaderless mRNA: *cI*(1–30)*lacZ* (1–684), leadered:(leader + RBS of pET22b)*cI*(1–30)*lacZ*(1–684)). Each mRNA was made *in vitro* using PCR products from pJW190 and pJW195 plasmids. *In vitro* translation experiment was performed with commercial *E. coli* S30 Extract System supplemented with [^35^S]-methionine. The mRNAs produced comparable amounts of 238-amino acid long (26 kDa) N-terminal CI-LacZ peptide independently of the presence of 5′-NAD on mRNA (Figure 5C). The overall yield of the peptides from the leadered mRNAs was higher, as expected.

**Figure 6.**
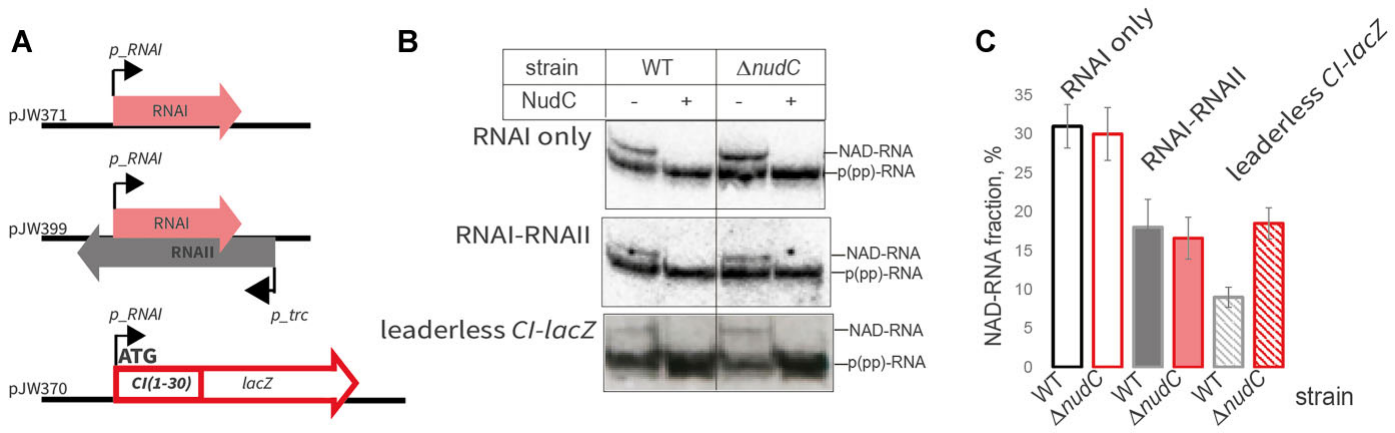
Dependence of RNA NADylation on duplex formation with antisense RNA and translation. (**A**) Scheme of the genes and regulatory elements on the otherwise identical plasmid used for the experiment. pJW371 expresses RNAI only, pJW399 expresses additionally RNAII from *p_trc* promoter, pJW370 contains chimeric leaderless ORF consisting of *lacZ* ORF fused with sequence coding for N-terminal 10 amino acid residues of CI protein of λ bacteriophage. (**B**) Northern blot probed against RNAI and mRNA *cI-lacZ*. Total RNA from WT/Δ*nudC* strains was treated/untreated with NudC and resolved on denaturing PAAG supplemented with 0.3% 3-acrylamidophenylboronic acid. For translated (leaderless) construct total RNA was first treated with DNazyme to produce shorter 5′ fragment of the *mRNA cI-lacZ*. (**C**) Plot of the fractions of NADylated RNAI produced in WT and *ΔnudC* strains. Values are mean ± SD from three biological replicates.

To test if leaderless 5′-NAD-RNA is translated in the cell, we constructed a plasmid vector with *lacZ* gene fused with a short initial portion of λ *cI* gene (coding for 10 N-terminal amino acid residues), placed under RNAI promoter in plasmid pJW370 (Figure 5C). This construct transformed into *E. coli* WT and Δ*nudC* strains produced comparable β-gal activity (Figure 5C). To confirm that this signal originates from protein translated from leaderless mRNA, we used Kasugamycin, an antibiotic specifically inhibiting leadered but not leaderless translation (52). We observed no significant difference in β-galactosidase activity from these two strains in the presence or absence of the antibiotic. The observation of ∼10% of NADylated RNA from this construct on Northern blot (Figure 6A, B), implies that NADylated mRNA is translated. This is because in *E. coli* transcription is coupled to translation, meaning that untranslated mRNAs are quickly degraded (53,54).

### The efficiency of NAD removal from NADylated RNA depends on its interacting partners

Although the effects can be sequence-specific, we have shown that 5′-NAD of model RNAI does not affect either its duplex formation with antisense RNA or its translation. We therefore wondered whether 5′-NAD can be protected by antisense RNA or ribosome binding, or alternatively exposed to degradation by the act of translation. We constructed plasmid vectors based on pACYC184 (p15A origin of replication) with either RNAI (from the pCDF plasmid with a colE1 origin) cloned under its own promoter, or both RNAI and 5′ -fragment of RNAII (under the strong constitutive *P*_trc_ promoter and a strong terminator upstream of *P*_RNAI_, Figure 6A). Note that fragment of RNAII is not able to initiate replication of the plasmid, since it is missing the priming part (33). As can be seen from Figure 6B, formation of duplex with RNAII slightly lowers down the proportion of NADylated RNAI, compared to the plasmid with RNAI only (Figure 6C). Notably, this proportion remains the same for Δ*nudC* strain, suggesting that the formation of RNA–RNA duplex does not affect processing of the RNAI by de-capping enzyme and further implying that the role of NudC might be limited to unstructured RNAs.

Translation, on the other hand, lowered the proportion of NADylated mRNA transcribed from RNAI promoter. In contrast to the negligible effect of NudC deletion on RNAI capping, a significant increase in capping was observed on model *cI-lacZ* mRNA transcribed from the same promoter (9% in WT versus 16.5% in Δ*nudC*). This may suggest that the absence of a secondary structure, caused by the translation process, exposes 5′-end to NudC de-NADing. Ribosome binding is perhaps too transient to protect the 5′-NAD of RNA.

### Processing of NADylated RNA-de-capping and processing by major RNases

One of the main functions of m^7^G cap is the regulation of mRNA stability (55). This function was also proposed for NAD-cap, but opposite effects for eukaryotes and bacteria were observed. While 5′-NAD in eukaryotes speeds up degradation (56), it was suggested to protect RNA in bacteria. This statement is based on one experiment where half-life of a modified RNAI was increased by 5′-NAD in *ΔnudC* strain (6).

**Figure 7.**
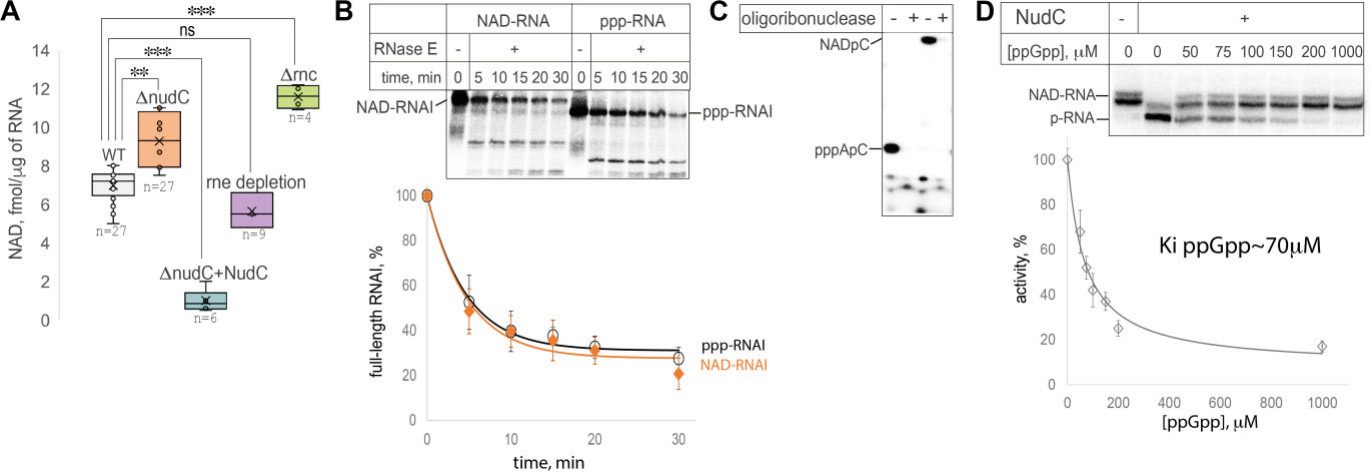
NADylated RNA processing with major nucleases and NudC. (**A**) Amount of NADylated RNA in cells with deletions and depletions of major RNases and de-capping enzyme NudC. Concentration of NAD on RNA was measured by FluorCapQ, Δ*rnc* is a deletion of RNaseIII, *rne* depletion is depletion of RNaseE at non-permissive temperature in *E. coli rne-3071* strain with thermosensitive RNaseE, Δ*nudC*+ *nudC* is a strain with deletion of *nudC* expressing NudC from a plasmid, n = number of biological replicates. *P*-values (two-sided Student's *t*-test) are shown above the histogram as in Figure 3B. (**B**) Kinetics of *in vitro* NAD- or ppp- RNA cleavage with RNaseE, values used for the plot are mean ± SD from three independent experiments. (**C**) Hydrolysis of dinucleotide NAD- and ppp-RNA with Orn oligoribonuclease. (**D**) Inhibition of NudC with ppGpp. NAD-RNA (the 5′ 70 nt portion of *cI*) was incubated with NudC at indicated concentrations of ppGpp. Below the gel is a plot of NudC activity vs [ppGpp], values are mean ± SD from three independent experiments.

The RNA degradation cascade in *E. coli* is initiated by RNaseE and finalized by oligoribonuclease Orn. The activities of these two essential enzymes are currently known to depend on the nature of an RNA 5′-end; there are no major 5′-dependent exonucleases in *E. coli*. We have not observed a significant change in the proportion of bulk NAD-RNA measured by FluorCapQ in a *E. coli* strain N3431 *rne*-3071(ts) (57) with temperature-sensitive RNaseE at non-permissive conditions, compared to WT (Figure 7A). The unchanged proportion of NAD-RNA upon RNaseE deactivation suggests that the degradation pathway based on RNase E is not a main pathway for the majority of NAD-RNA.

RNaseE acts via two alternative mechanisms—5′-dependent and 5′-independent ones (58). It is well-documented that for RNAI the first degradation step is 5′-end proximal cleavage by RNaseE (59). Therefore, NAD at 5′-end of RNA could affect the efficiency of processing by RNaseE. However, consistently with the *in vivo* results, *in vitro* we observed that NAD-RNAI and ppp-RNAI degradation kinetics by RNaseE are the same (Figure 7B).

In contrast to RNaseE, RNaseIII deletion caused the accumulation of NAD-RNA, as measured by FluorCapQ (Figure 7A). Together these results suggest that NAD-RNA is preferentially degraded by RNaseIII, endonuclease acting on RNA duplexes, in agreement with the estimation that most NAD-RNA species are structured sRNA, of which many are shown to form duplex with antisense RNAs as part of their regulatory mechanism (1).

The final step of RNA degradation is carried out by the essential enzyme oligoribonuclease (Orn), the only enzyme in *E. coli* capable of breaking down RNA fragments shorter than five nucleotides into mononucleotides (60). Would 5′-NAD present a barrier for degrading 5′-NADylated dinucleotides? Using dinucleotides bearing either 5′-ppp- or 5′-NAD (produced by *E. coli* RNAP *in vitro* abortive synthesis on RNAI promoter fragment), we found that Orn can degrade completely both NADpC and pppApC dinucleotides (Figure 7C). This result suggests that 5′-NAD does not inhibit the activity of Orn nuclease.

Levels of NADylated RNA would depend on NudC de-capping, since it would create monophosphorylated 5′-end, sensitive to RNase E degradation (17). However, we found that NudC has only a modest effect on NADylated RNA levels *in vivo*, judging from bulk FluorCapQ assay (Figure 7A) as well as RNAI deNADing visualized by northern blotting (Figure 6B). However, when NudC was overexpressed from a plasmid vector, it resulted in almost complete removal of NAD-RNA (Figure 7A). This implies that either the levels of NudC or its catalytic rate are relatively low under standard conditions, indicating the potential for regulation of de-capping.

In a few publications, it has been noted that NADylated RNA levels are higher in the stationary phase (7,61). Likewise, in our hands, levels of NAD-RNA are slightly but reproducibly higher in stationary phase, yet levels of NAD or competing substrate ATP do not change much in the stationary phase. (Supplementary Figure S2). Therefore, to explain higher bulk NAD-RNA in these conditions (as published e.g. in Bird *et al.* (6); our data in Supplementary Figure S2) we assumed that the levels of NudC to drop during stationary phase. However, contrary to the expectations, the published transcriptomics data show that levels of *nudC* transcript actually rise in the stationary phase and are not affected by anaerobic growth conditions (Supplementary Figure S5, source data Reigstad *et al.* (62)), where we observed higher bulk NADylation of RNA (Figure 1A).

We hypothesize that at least partially the observed effect could be due to less efficient NAD removal by NudC in the stationary phase, presumably caused by its inhibition by an external factor. This hypothesis is supported by reports from high-throughput proteomics studies that NudC binds ppGpp (63). Indeed, *in vitro* we found that NudC activity is inhibited by ppGpp with inhibition constant in the region of ∼70 μM, which is in physiological range of ppGpp concentration (Figure 7D).

## Discussion

Here, we broadly examined the physiological conditions that influence RNA NADylation and the effects of 5′-NAD on RNA fate and translation.

At the level of incorporation in accordance with *ab initio* mechanism, 5′-NADylated RNA levels follow an absolute intracellular NAD concentration. However, this concentration (∼220 μM) remains below apparent *K*_M_ for NAD as a transcription initiation substrate (∼400 μM) at tested conditions.

The only efficient way to raise the levels of RNA NADylation we found is to lower the concentration of ATP, the competing initiation substrate. This requires extreme conditions bacteria rarely encounter or survive, such as membrane depolarisation/ proton gradient dissipation. Even then NADylation remains a minor modification e.g. reaching 35% for the RNAI and 36% for SibD transcripts, species reported as most highly NADylated (1,7). Therefore, it seems unlikely to come across a physiological state at which a given transcript could be fully NADylated, at least in *E. coli*. Interestingly, Rifampicin treatment may create *E. coli* variants more prone to use alternative initiating substrates, as the preference for NAD can be increased e.g. by R529H, rifampicin-resistant change in β subunit. It also suggests that RNAPs of other species with different architecture of the active site could be more prone to NAD incorporation.

DNA supercoiling is another potential factor stimulating NAD incorporation, perhaps aiding promoter DNA melting and supporting ability of 5′-NAD to stabilize short transcripts (5). Supercoiling may also contribute to the higher NADylation seen in stationary phase (7,64). During stationary phase, *E. coli* chromosome displays a gradient of negative supercoiling, with a maximum near the terminus. This gradient is lost in exponential phase (65). In our bulk approach experiments, we examined total cellular NAD capping. Therefore, it might be possible that the higher level of NAD capping during the stationary phase originates from genes which are localized within the locally supercoiled parts of the bacterial chromosome. It also suggests that the relatively weak consensus motif found in promoters predisposed for NAD incorporation (61) may reflect not the sequence requirement but the propensity to assume a favourable DNA conformation. For plasmid DNA the supercoiling is lower in stationary phase (66), meaning that other factors may play role in increasing NADylation of plasmid-encoded RNAs.

We found that *E. coli* 70S ribosome does not distinguish between AUG and non-canonical NAD-UG start codons, and that leaderless NAD-RNA is translated *in vivo*. *E. coli* codes for only a few leaderless mRNAs, mostly originating from prophages. However, there are species with a high proportion of leaderless transcripts, including *Thermus/Deinococcus* (up to 40%), Archaeal (∼30%) and *Mycobacterial* (∼15%) clades (20). Potentially in these clades NADylation may have a more pronounced effect on translation.

We suggest that NADylation differentially affects the processing of sRNA and mRNA due to the NudC preference for the exposed 5′-NAD of RNA (16). Structured sRNAs commonly act via annealing with an antisense target. We showed that base-pairing kinetics and affinity of NADylated RNA to its partner are not affected, at least for RNAI-RNAII pair. Once 5′-NAD-RNA forms a duplex whether *in cis* or *in trans*, it becomes resistant against processing by NudC. This scenario, considering that bulk NADylated transcripts are sRNAs, is supported by the very modest effect of NudC and the noticeable effect of RNaseIII (endonuclease insensitive to the nature of the RNA 5′-end (67)) on steady-state NADylation levels of bulk RNA and selected sRNA species. Perhaps the processing pathway might be more complex and affected by 5′-NAD modification when the base-pairing of sRNA with its target is short and imperfect, and if sRNA is a target for Hfq chaperone.

The outcome of 5′-NADylation for mRNA seems to be different. We showed that the process of translation exposes 5′-NAD for de-capping by NudC and subsequent RNA degradation. This mechanism, perhaps mostly relevant for mRNAs with relatively short 5′ untranslated regions (and leaderless) may explain why translated NADylated RNAs are a minority compared to non-translated ones found so far (1). Ribosome binding seems too transient to protect the 5′-NAD of mRNA. Therefore, it is possible that more mRNAs are NAD-capped at the stage of transcription initiation and later rapidly processed.

NudC catalytic activity appears to have a crucial impact in determining the fate of NADylated RNA. Could changes in levels of NudC or its regulation account for the connection of NADylation to the cell's physiological state, e.g. NADylation increase in the stationary phase? Published data, consistent with our findings, showed that counterintuitively, both *nudC* expression and NAD-RNA levels are increased in the stationary phase (Supplementary Figure S5, (7)). This paradox might be explained by our discovery that ppGpp inhibits NudC, with an inhibition constant of approximately 70 μM. This concentration falls within the physiological range of ppGpp concentration—around 100 μM during the stationary phase and transiently higher during the transition from exponential to stationary phase (68). If there are inhibitors of NudC, it is plausible that there are also activators. Potentially, NudC might serve a specific function under unique conditions, such as during phage infection, as recent work of Wolfram-Schauerte *et al.* suggests yet unknown role of NAD-RNAs by their attachment to the protein targets of viral ADP- ribosylation (69). Apart from NudC-related regulation, we cannot exclude some yet unknown conditions favouring e.g. degradation of 5′-triphosphorylated transcripts.

NAD modification of RNA seems to exist largely unnoticed by *E. coli* RNA processing and translation machinery, at least in a range of laboratory conditions tested here. This neutrality is a valuable feature for potential future synthetic biology applications of 5′-NAD RNA. Our work points towards a conceivably higher role of 5′-NAD RNA in species characterized by high intracellular NAD levels, potentially those that grow anaerobically and have a high proportion of leaderless mRNA.

Overall, our findings suggest that NADylation occurs as an incidental RNA modification due to the inherent promiscuity of RNAP at the initiation stage. Early research has long recognized that RNAP can accidentally incorporate diverse non-canonical substrates, such as dinucleotides, during initiation (70,71). We can further speculate that if the specific structure of the 5′-end of RNA had a significant (negative) impact on vital functions, the permissiveness of transcription initiation in bacteria would likely have been altered during their evolutionary history.

## Supplementary Material

gkae813_Supplemental_File

## Data Availability

The data underlying this article are available in the article and in its online supplementary material.
