# Supplementary material for: Stochastic nature and physiological implications of 5′-NAD RNA cap in bacteria
Source: Nucleic Acids Res. 2024 Sep 26;52(19):11838–52. doi: 10.1093/nar/gkae813 (PMC11514452; doi:10.1093/nar/gkae813)
Supplement: gkae813_Supplemental_File [file gkae813_supplemental_file.pdf]

Supplementary Information

FluorCapQ, a new method for detection and quantification of NAD modification on RNA.

Some of the currently used methods for 5'-NAD RNA quantification are either not directly quantitative based on NGS, or require labour-intensive LC/MS for broad screening of different mutants and conditions (1).

The most used method for quantification of 5'-NAD of RNA, NAD-capQ(2), is based on NAD quantification of nuclease P1-treated total RNA samples by commercial NAD quantification kit (NAD/NADH Quantitation Kit, Sigma-Aldrich). Nuclease P1 hydrolyses RNA to mononucleotides and releases free NAD, which is sensed by the colorimetric assay and measured by absorbance at 450 nm (A_{450}) according to the kit manufacturer's protocol. NAD bound to RNA (untreated with nuclease P1) should not cause a colorimetric change in measured samples and serves as a background control.

We reproduced the method and found out that the obtained values of NAD capping are dependent on the RNA extraction method and the ratio of input cell mass to TRIzol reagent: the higher the ratio, the higher the detected NAD content per mg of isolated RNA (Supplementary figure 1A). The authors(2) recommended to use 1 ml of TRIzol reagent per 17 OD units of bacterial culture, contrary to the manufacturer recommendation. Even when subtracting the P1-untreated background, the NAD concentration values correlate with the number of cells used for RNA isolation (Supplementary figure 1B), suggesting a contamination with free NAD. When an extra purification step is added (Monarch RNA clean-up column purification) the amount of NAD capping drops below the detection limit of the colorimetric method for NAD quantification (Supplementary figure 1A).

The benefit of our new method FluorCapQ is that it does not require RNA treatment by nuclease P1 (Main Figure 1B), which is a crucial step for NAD quantification by NAD-capQ(2). Nuclease P1(3) is a single-strand specific nuclease which hydrolyses phosphodiester bonds in RNA and DNA. Any RNA secondary structure at its 5' end can hinder the NAD release and potentially affect the NAD quantification with NAD-capQ. While many of the identified NAD-capped RNAs are sRNAs(4,5), which can be highly structured, the requirement for nuclease P1 step can affect the measurement. Direct fluorescence measurement of capped RNA omits the enzymatic step and, therefore, prevents any potential bias of nuclease P1 hydrolytic efficiency.

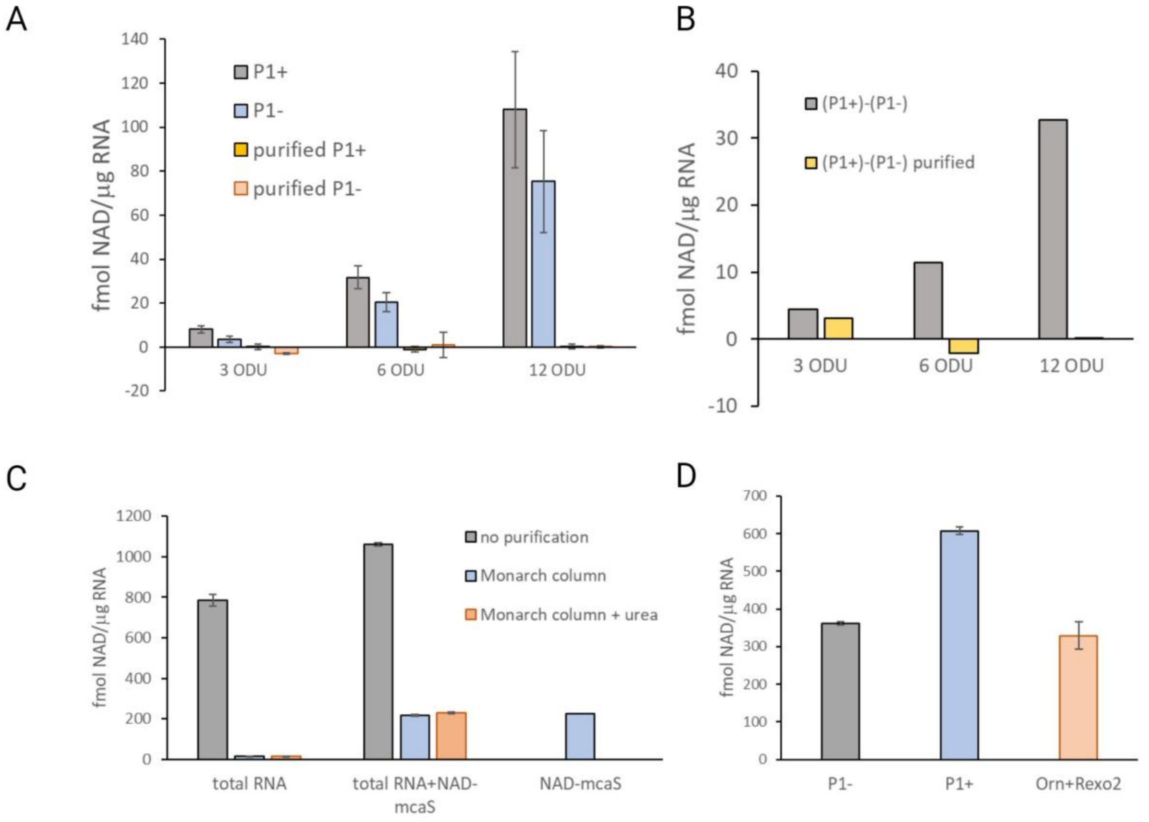

Supplementary Figure 1. Importance of removing free NAD from RNA samples. **A.** NADcapQ quantification of NAD in total RNA samples isolated using 1 ml TRIzol per various amounts of *E. coli* cells (3, 6 and 12 OD units; ODU). Samples were or were not treated with nuclease P1 as described before (1) and further purified by Monarch RNA clean-up column before the NAD quantification when stated. Error bars represent the standard deviation calculated from 3 biological replicates. **B.** NAD quantification of **A.** after the P1 untreated background being subtracted; (P1+)-(P1-). **C.** NAD content in samples was quantified by FluorCapQ after the combination of various purification steps (sample purification with Monarch RNA clean-up column (New England Biolabs) pretreated or not by heating in 2M urea for 2 min at 65°C). The input RNAs were unpurified bacterial total RNA (10 mg) and *in vitro* transcribed, Monarch column-purified NAD-mcaS (93 nt long, 7 ng) in different combinations; error bars represent the standard deviation calculated from 3 technical replicates. **D.** Oligoribonucleases Orn and Rexo2 do not release a significant amount of free NAD. Bacterial total RNA (50 mg) was or was not treated with nuclease P1 or with a combination of oligoribonucleases Orn and Rexo2. The free NAD was quantified by NADcapQ. Error bars represent the range of 2 technical replicates.

The extra purification step during RNA extraction does not allow free NAD contamination to affect FluorCapQ

During the process of RNA purification as recommended for NAD-capQ (TRIzol extraction, precipitation with sodium acetate, heating in buffer containing 2 M urea and precipitation with ammonium acetate(2)), various amounts of free NAD could be copurified with the total RNA (Supplementary Figures 1A, 1B). To lower the amount of potentially non-covalently attached NAD, we performed a subsequent purification of RNA by Monarch RNA clean-up kit, using the manufacturer-adjusted protocol, enabling efficient recovery of RNA ≥ 15 nt (see the manufacturer's protocol). This step reproducibly lowered the quantified NAD from hundreds to several fmols per mg of total RNA. We verified that, unlike the free NAD, the model NAD-RNA of sRNA size (around 100 nt), NAD-mcaS is not being lost during this purification step. We mixed un-purified total RNA with *in vitro*-prepared NAD-mcaS and compared the NAD quantity before and after several purification methods, including column purification and heating in 2 M urea (Supplementary Figure 1C). We found that Monarch column clean-up is sufficient to remove free NAD and to keep NAD-capped RNA.

The only concern was whether the loss of the signal after purification comes from the loss of free NAD or NAD-capped oligonucleotides shorter than 15 nt-column cut-off. Therefore, we used the NAD capQ method to quantify NAD released by nuclease P1 and by oligoribonucleases (Orn and Rexo2). If the signal loss after Monarch column was from NAD-oligoribonucleotides, we should have seen an increase of free NAD after Orn and Rexo2 treatment, which was not the case (Supplementary Figure 1D). We conclude that the Monarch column purification removes just free NAD. The ability of Orn and Rexo2 to release NAD from NAD-oligoribonucleotides is demonstrated in Fig. 6C in the main text.

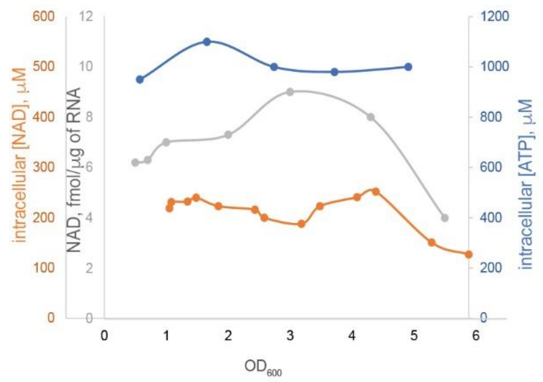

Supplementary Figure 2. Levels of ATP (blue curve and axis), NAD (orange curve and axis) and a bulk NADylated RNA (grey curve, black axis) during *E. coli* growth in LB. Representative plots are shown without SD; experiments were performed ≥3times with similar results.

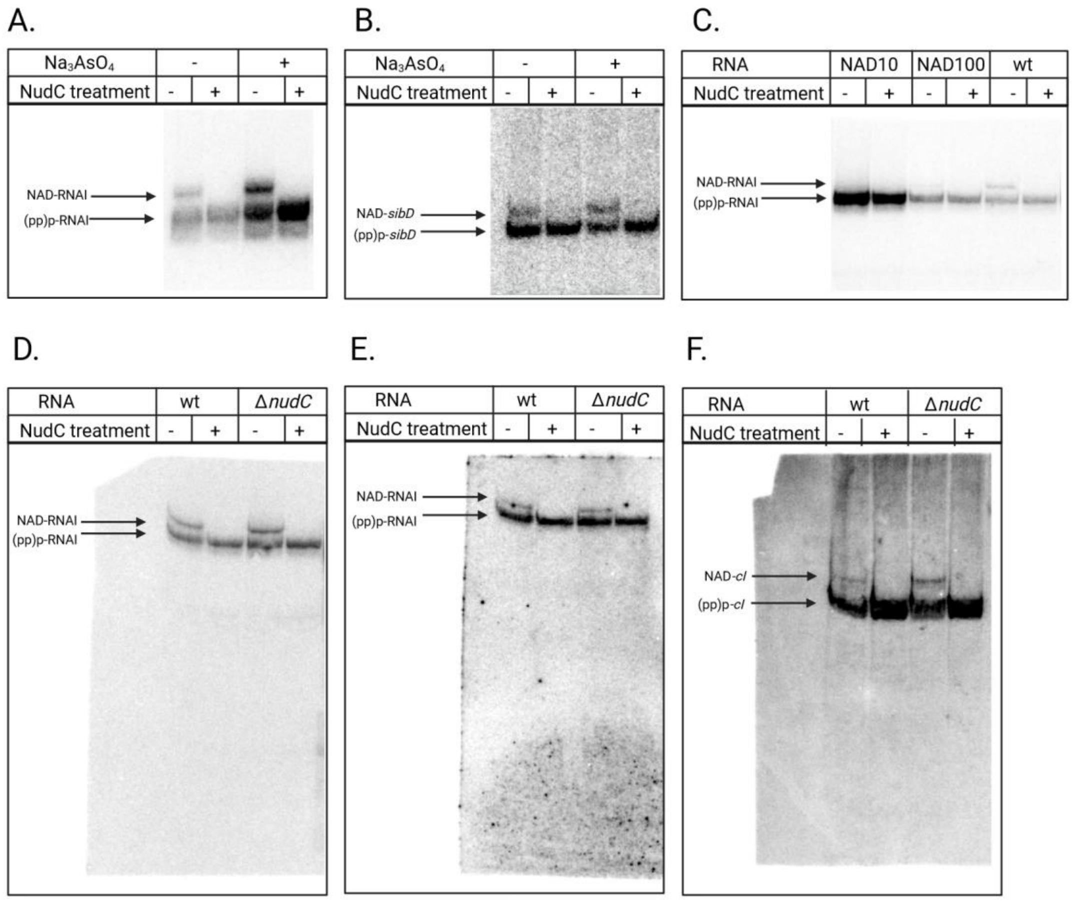

Supplementary Figure 3. Representative Northern blots used for cutouts in Figures 2C and 6B.

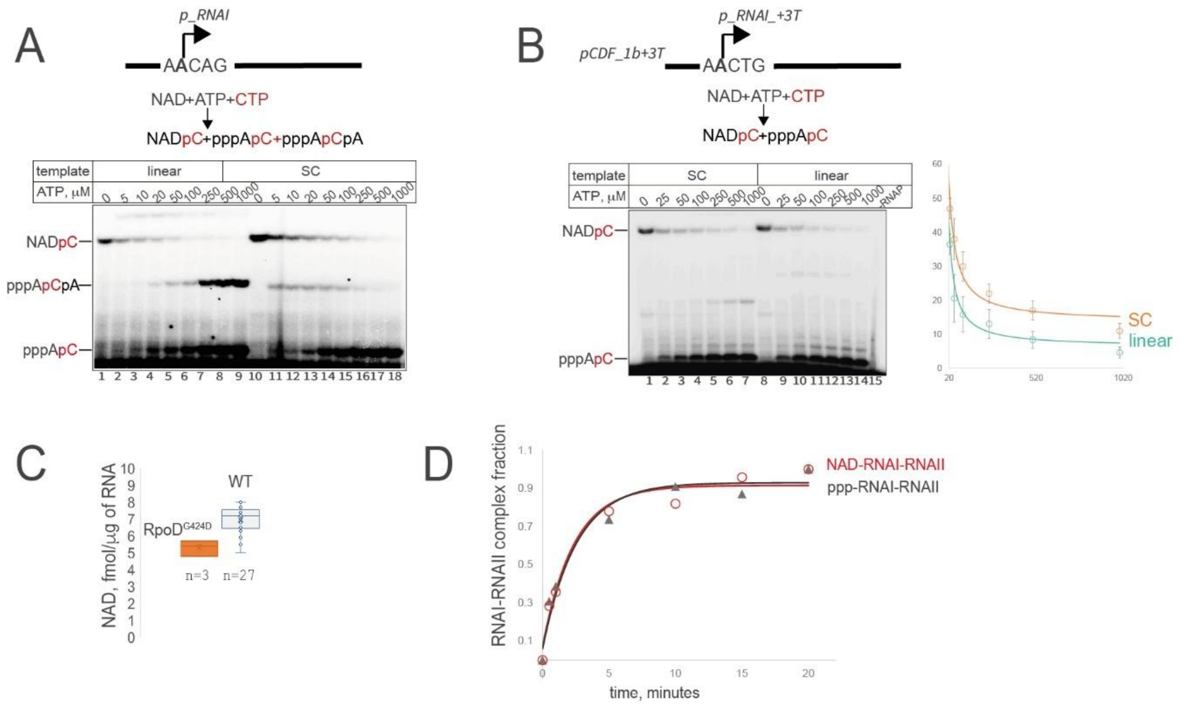

Supplementary Figure 4. A. *In vitro* transcription initiation with NAD on supercoiled plasmid template is more efficient compared to linearized plasmid pCDF-1b template. The efficiency of initiation was assessed using increasing concentrations of ATP at stable 250 µM NAD, on supercoiled (SC) and linear plasmid template. The scheme of experiment with sequence of the template around transcription start site, substrates and products of reaction are shown above the gel. **B**. ***In vitro* transcription initiation with NAD on supercoiled plasmid template is more efficient compared to linearized plasmid pCDF-1b_+3T template.** The efficiency of initiation was assessed using increasing concentrations of ATP at stable 250 µM NAD, on supercoiled (SC) and linear plasmid template. The scheme of experiment with sequence of the template around transcription start site, substrates and products of reaction are shown above the gel. Right: the plot shows results of the competition experiment using supercoiled and linear templates. Data were fit to y=y0+(a*b)/(b+x) equation. Values are mean ± SD from three independent experiments. **C.** Stringent initiation factor σ^{G424D}decreases the amount of NADylated RNA. **D.** Kinetics of NADylated RNAI binding to RNAII is the same as triphosphorylated RNAI.

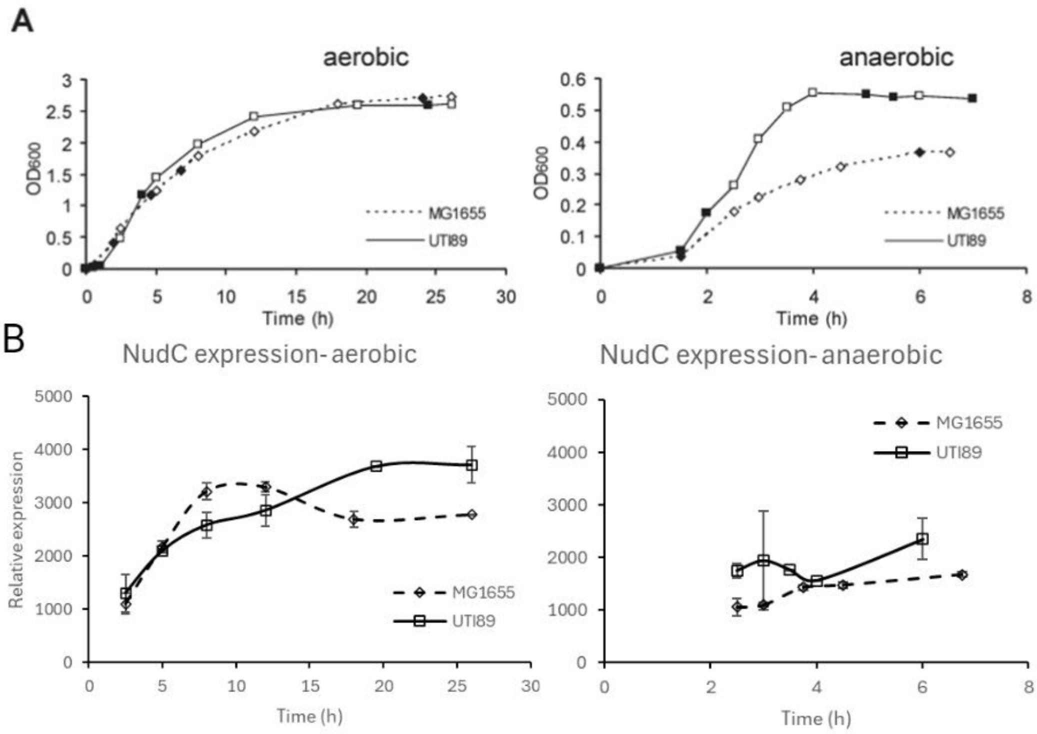

Supplementary Figure 5. Transcriptional expression of NudC in MG1655 and UTI89 *E.coli* cells harvested from various time points during aerobic or anaerobic growth in Luria-Bertani medium (source (6) GEO accession number GSE6425). **A.** Growth curves of *E.coli* MG1655 and UTI89 under aerobic and anaerobic conditions. **B**. Expression profile of *nudC* from different growth phases (RNA isolated at time points 2.5, 5, 8, 12, 18, 26 h during aerobic growth and at 2.4, 3, 3.75, 4.5, 6.75 h during anaerobic growth).

Supplementary Table 1. Levels of NAD-RNA in strains with mutations in Rifampicin binding pocket of RNAP. The values are mean values ± SD from three biological replicates

Strain	NAD-RNA, fmol/µg of total RNA
WT	7.0 ± 1.8
RpoB S512F	8.6 ± 2.8
RpoB Q513L	5.8 ± 1.8
RpoB P514C	7.5 ± 1.5
RpoB H526Q	6.6 ± 1.5
RpoB R529H	14.9 ± 2.4
RpoB I572F	6.1 ± 1.5

Supplementary Table 2. Bacterial strains

Strain number	Strain name	Aditional details	Source
JW221	WT *E. coli*	K-12 BW25113	*E. coli* Genetic Stock Center, Yale University
JW222	Δ*nudC*, KEIO JW5548-1	F-, Δ(*araD*-*araB*)567, Δ*lacZ*4787(::*rrnB*-3), λ-, *rph*-1, Δ(*rhaD*-*rhaB*)568, Δ*nudC*767::*kan*, *hsd*R514	KEIO collection (7)
Yje004 (JW335)	NAD auxotroph	Yje004= BW25113/pET15K-*NTT4*/*nadD*::*cat*	(8)
rpoB(S512F)	S512F	*rpoB*(S512F)	This work
rpoB(Q513L)	Q513L	*rpoB*(Q513L)	This work
rpoB(H526Q)	H526Q	*rpoB*(H526Q)	This work
rpoB(R529H)	R529H	*rpoB*(R529H)	This work
rpoB(I572F)	I572F	*rpoB*(I572F)	This work
KEIO JW1225-2	Δ*hns*, KEIO JW1225-2	F-, Δ(*araD*-*araB*)567, Δ*lacZ*4787(::*rrnB*-3), λ-, Δ*hns*-746::*kan*, *rph*-1, Δ(*rhaD*-*rhaB*)568, *hsd*R514	(7)
KEIO JW1225-2 +hns	pET28 *hns in* KEIO JW1225-2	BW25113 pET28:PT7 lacO *hns* (overexpression of HNS; T7 promoter, IPTG inducible)	This work
KEIO JW5808-1	Δ*pcnB*, KEIO JW5808-1	F-, Δ(*araD*-*araB*)567, Δ*pcnB*759::*kan*, Δ*lacZ*4787(::*rrnB*-3), λ-, *rph*-1, Δ(*rhaD*-*rhaB*)568, *hsd*R514	(7)
MG1655 ΔrpoD p_rpoD^{G424D}	G424D	*rpoD* G424D	Prof David Grainger, University of Birmingham, (9)
Hfr(PO1)	*rne* thermosensitive	strain *Hfr*(PO1), *lacZ*43(Fs), λ-, *rne*-3071(ts)	Prof Ben Luisi, Cambridge University
JW342	Δ*nudc*+pCA24N *nudC*	JW222 with *nudC* under $P_{T5\text{-}lac}$ promoter	This work
AB301-105	Δ*rnc*	Δ*rnc* strain AB301-105	*E. coli* Genetic Stock Center, Yale University
JW387	JW370 in wt	plasmid pJW370 in JW221	This work
JW389	JW370 in Δ*nudC*	plasmid pJW370 in JW222	This work
JW375	pJW371 in wt	plasmid pJW371 in JW221	This work
JW377	pJW371 in Δ*nudC*	plasmid pJW371 in JW222	This work
JW381	pJW371 in NAD auxotroph	plasmid pJW371 in Yje004	This work
JW395	JW399 plasmid in WT	plasmid pJW399 in JW221	This work
JW396	JW399 plasmid in Δ*nudC*	plasmid pJW399 in JW222	This work

JW398	JW399 plasmid in Yje004	plasmid pJW399 in Yje004	This work

Supplementary Table 3. Plasmids

Plasmid number	plasmid name	plasmid description	Source
pCDF-1b	pCDF-1b	Bacterial vector with a CloDF13 (CDF) origin and spectinomycin/streptomycin resistance gene	Novagen
pACYC184	pACYC184	Bacterial vector with a p15A origin of replication and a tetracycline resistance gene	ATCC
pCA24N nudC	pCA24N *nudC*	NudC under $P_{T5\text{-}lac}$ promoter for expression in *E. coli*	ASKA collection, strain JW5548 (10)
pJW195	*cI-lacZ* in pCDF leaderless	pCDF-1b: P_{RM} *cI*(1-30) lacZ	This work
pJW190	*cI-lacZ* in pCDF leadered	pCDF-1b: P_{RM} (leader+RBS from pET22b) *cI*(1-30) lacZ	This work
pJW370	pACYC184-c*I-lacZ*	pACYC184: P_{RNAI} *cI*(1-30) *lacZ*	This work
pJW371	RNAI only	pACYC184: P_{RNAI}RNAI(pCDF-1b)	This work
pJW399	RNAI: P_{trc}RNAII	pACYC184: P_{RNAI}RNAI(pCDF-1b), antisense P_{trc} RNAII(1-128) T7 terminator	This work
pET28 *nudC*	pET28 *nudC*	pET28:P_{T7} *lacO nudC* (overexpression of NudC; T7 promoter, IPTG inducible)	This work
pET28 *orn*	pET28 *orn*	pET28*:*P_{T7} *lacO orn* (overexpression of Orn; T7 promoter, IPTG inducible)	This work
pCDF-1b_+3T	pCDF-1b_+3T	pCDF-1b RNAI +3A to T	This work

Supplementary Table 4. Oligonucleotides

Primer number	primer name	primer sequence
W1	t7 cI for	GATCCGAATAATACGACTCACTATTATGAGCACAAAAAAGAAAC
W2	t7 cI rev	CATAAATTGCTTTAAGGCGACGTGCGTCCTC
W10	cI rev 5'bio	5' Biotin-TCAGCCAAACGTCTCTTCAG
W32	antiCloDF13 3'bio	TTATGAGCCCGACGAGCTACCAGGCT-Biotin 3'
JW38	probe anti cI	TTGTGTTAATGGTTTCTTTTTTGTGCTCATACG
W100	t7 cI original 70nt	GATCCGAATAATACGACTCACTATTATGAGCACAAAAAAGAAACCATTAACACAAGAGCAGCTTGAGGACGCACGTCGCCTTAAAGCAATTTATG
W114	t7 leadered 25kDa for	GATCCGAATAATACGACTCACTATTACTGTAGAAATAATTTTGTTTAACTTTAATAAGG
W115	t7 leadered 25kDa rev	AATCATCCGCCACATATCCTG
W116	t7 leaderless 25kDa for	GATCCGAATAATACGACTCACTATTATGAGCACAAAAAAGAAACCATTAAC
W131	dnazyme targeting 5' end of lacZ	GTTGGGTAAGGCTAGCTACAACGAGCCAGGGT
JW191	probe anti sibD	TTCTTCGGGAGGGGCTTTCC
JW 198	probe anti RNAI	CGCAGAGCACAGCAACCAAATCTG
W220	rnaII t7 rev	AGTCCGGCTACACTGGAAG
W222	rnaI t7 rev	AACGAAAAAACCACCTGGG
W238	rnaI t7 for polyA	TTTTTTAACGAAAAAACCACCTGGG
W239	rnaI t7 for	GATCCGAATAATACGACTCACTATTACAGATTTGGTTGCTGTGC
W240	rnaII t7 for	GATCCGAATAATACGACTCACTATAGTAAACGAAAAAACCACCTGG
W253	anti sibD t7 for probe	GATCCGAATAATACGACTCACTATTGGAAAGCCCCTCCCGAAGAA
W254	anti sibD t7 revprobe	ACAAGGGTGAGGGAGGATTTCTC

References

1. Chen, Y.G., Kowtoniuk, W.E., Agarwal, I., Shen, Y. and Liu, D.R. (2009) LC/MS analysis of cellular RNA reveals NAD-linked RNA. *Nat Chem Biol*, **5**, 879-881.
2. Grudzien-Nogalska, E., Bird, J.G., Nickels, B.E. and Kiledjian, M. (2018) "NAD-capQ" detection and quantitation of NAD caps. *RNA*, **24**, 1418-1425.
3. Lahm, A., Volbeda, A. and Suck, D. (1990) Crystallisation and preliminary crystallographic analysis of P1 nuclease from Penicillium citrinum. *J Mol Biol*, **215**, 207-210.
4. Cahova, H., Winz, M.L., Hofer, K., Nubel, G. and Jaschke, A. (2015) NAD captureSeq indicates NAD as a bacterial cap for a subset of regulatory RNAs. *Nature*, **519**, 374-377.
5. Zhang, H., Zhong, H., Wang, X., Zhang, S., Shao, X., Hu, H., Yu, Z., Cai, Z., Chen, X. and Xia, Y. (2021) Use of NAD tagSeq II to identify growth phase-dependent alterations in E. coli RNA NAD(+) capping. *Proc Natl Acad Sci U S A*, **118**.
6. Reigstad, C.S., Hultgren, S.J. and Gordon, J.I. (2007) Functional genomic studies of uropathogenic Escherichia coli and host urothelial cells when intracellular bacterial communities are assembled. *J Biol Chem*, **282**, 21259-21267.

7. Baba, T., Ara, T., Hasegawa, M., Takai, Y., Okumura, Y., Baba, M., Datsenko, K.A., Tomita, M., Wanner, B.L. and Mori, H. (2006) Construction of Escherichia coli K-12 in-frame, single-gene knockout mutants: the Keio collection. *Mol Syst Biol*, **2**, 2006 0008.
8. Zhou, Y., Wang, L., Yang, F., Lin, X., Zhang, S. and Zhao, Z.K. (2011) Determining the extremes of the cellular NAD(H) level by using an Escherichia coli NAD(+)-auxotrophic mutant. *Appl Environ Microbiol*, **77**, 6133-6140.
9. Lamberte, L.E., Baniulyte, G., Singh, S.S., Stringer, A.M., Bonocora, R.P., Stracy, M., Kapanidis, A.N., Wade, J.T. and Grainger, D.C. (2017) Horizontally acquired AT-rich genes in Escherichia coli cause toxicity by sequestering RNA polymerase. *Nat Microbiol*, **2**, 16249.
10. Kitagawa, M., Ara, T., Arifuzzaman, M., Ioka-Nakamichi, T., Inamoto, E., Toyonaga, H. and Mori, H. (2005) Complete set of ORF clones of Escherichia coli ASKA library (a complete set of E. coli K-12 ORF archive): unique resources for biological research. *DNA Res*, **12**, 291-299.
